# Modulating D-amino acid oxidase (DAAO) substrate specificity through facilitated solvent access

**DOI:** 10.1371/journal.pone.0198990

**Published:** 2018-06-15

**Authors:** Kalyanasundaram Subramanian, Artur Góra, Ruud Spruijt, Karolina Mitusińska, Maria Suarez-Diez, Vitor Martins dos Santos, Peter J. Schaap

**Affiliations:** 1 Laboratory of Systems and Synthetic Biology, Wageningen University & Research, Stippeneng WE, Wageningen, The Netherlands; 2 Tunneling Group, Biotechnology Centre, Silesian University of Technology, ul. Krzywoustego, Gliwice, Poland; 3 Department of Chemistry, Silesian University of Technology, ks. Marcina Strzody, Gliwice, Poland; Universidade Nova de Lisboa Instituto de Tecnologia Quimica e Biologica, PORTUGAL

## Abstract

D-amino acid oxidase (DAAO) degrades D-amino acids to produce α-ketoacids, hydrogen peroxide and ammonia. DAAO has often been investigated and engineered for industrial and clinical applications. We combined information from literature with a detailed analysis of the structure to engineer mammalian DAAOs. The structural analysis was complemented with molecular dynamics simulations to characterize solvent accessibility and product release mechanisms. We identified non-obvious residues located on the loops on the border between the active site and the secondary binding pocket essential for pig and human DAAO substrate specificity and activity. We engineered DAAOs by mutating such critical residues and characterised the biochemical activity of the resulting variants. The results highlight the importance of the selected residues in modulating substrate specificity, product egress and enzyme activity, suggesting further steps of DAAO re-engineering towards desired clinical and industrial applications.

## Introduction

D-amino acid oxidase (DAAO) catalyses the dehydrogenation of nonpolar and hydrophobic D-amino acids (EC 1.4.3.3) [1]. Previous investigations in yeast and mammalian DAAOs have elucidated copious details on the reaction mechanism using structural characterization [1–9]. Residues in the first shell of DAAO’s active site position the substrate against the mandatory cofactor, flavin adenine dinucleotide (FAD), which is noncovalently bound to DAAO. The cofactor FAD receives hydrogen from the substrate to produce an intermediate imino acid and hydrogen peroxide. The imino acid spontaneously deaminates to its respective α-ketoacid and releases ammonia. Oxygen channels bring molecular oxygen into the active site, which reoxidizes FAD at the end of the reaction cycle [10,11]. Despite the presence of water molecules in the active site that assist in hydrogen peroxide production, the hydrophobic environment around FAD is imperative for the substrate oxidation step [1,5,6,8,10,12].

Many similarities can be identified among DAAOs from yeast (*Trigonopsis variabilis*, TvDAAO, and *Rhodotorula gracilis*, RgDAAO) and mammals (pig, pkDAAO, and human, hDAAO). Notwithstanding their overall structural similarity, yeast DAAOs differ from the mammalian DAAOs in their stability, activity, the rate-limiting step and substrate specificity [1,2,4,13]. Both pkDAAO and hDAAO possess an ‘active site loop’ that functions as the ‘lid’ of the funnel-shaped entrance to the active site [1–3,6,9,12–15]. The active site plasticity of both pkDAAO and hDAAO is the result of the remarkable conformational dynamics exhibited by the lid loop, which consists of a key residue (Y224) that functions as a gate [12,16] and is an essential residue for DAAO activity. The lid loop movement regulates access to the active site, inhibitor/substrate specificity and provides the hydrophobic environment required around FAD. The slow conformational switching of the lid loop and Y224 from the closed to open position influences product release, which defines the rate-limiting step of pkDAAO and hDAAO [1,2,9,10,14,17,18].

Besides the active site, pkDAAO and hDAAO are equipped with a hydrophobic secondary binding site, whose entrance is lined by hydrophobic residues. The secondary binding site, in particular, influences the orientation/specificity of the substrate/inhibitor in mammalian DAAOs [1–3,5,6,16–19]. Yeast DAAOs, which lack the sophisticated features of pkDAAO and hDAAO, such as the dynamics of the lid loop and the Y224 gate, as well as a well-defined secondary binding pocket, have FAD reduction as the rate-limiting step.

Many intensive engineering investigations have been performed on yeast and mammalian DAAOs [1,2,9,14,15,20–25] by mutating multiple residues (including those located in various loops) to investigate possible modifications of functional properties. The activity of DAAO has been shown to be essential for human health. Therefore, DAAO-related substrates, analogues, metabolites, and inhibitors have been proven to be valuable, not only for industrial and biotechnology purposes but also in pharmaceutical applications [26–39]. However, the complete mechanisms of product release, substrate specificity modulation and activity variation among DAAOs remain unclear.

Growing evidence highlights the importance of access pathways and gates as crucial features regulating the activity and substrate specificity of enzymes with a buried active site [40–43]. Since pkDAAO and hDAAO have a buried active site and product egress is their rate-limiting step, we concentrated on the initial analysis on their active sites, secondary binding sites and surroundings [3,4]. Solvent access investigations presented substantial details and identified critical residues, which were congruent with available literature data on (i) the structure and functional characterization of DAAOs [1–24,28–37], (ii) the analysis of the dynamics of the D-amino acid oxidase–benzoate complex of pkDAAO [44] and (iii) the aromatic cage regulating active site access and modulating the features of monoamine oxidase (MAO) [45].

Over 50 pkDAAO and hDAAO variants were designed and constructed, and their activity and substrate specificity were characterized using a high-throughput method [46]. These results were further combined with an analysis of the solvent access pathway. Finally, hDAAO wild-type Y55A and Y55A-L56T variants were select for a detailed computational study of their dynamics. The biochemical activity of these variants towards 19 D-amino acids and glycine substrates was measured.

Our results show that by facilitating solvent and substrate access, we were able to modulate the substrate specificity of pkDAAO and hDAAO. As a result, we have designed, constructed and characterized variants that extend the industrial, biotechnology and clinical applications of DAAO.

## Results

### Initial mutant selection and variants design

We focused on those DAAO residues that might regulate substrate access and/or product release. We included residues that otherwise remained so far as non-obvious choices for engineering investigations. Therefore, besides residues in the first and second shell of the pkDAAO active site, residues located in the vicinity of the lid loop of pkDAAO and hDAAO were selected. We designed pkDAAO and hDAAO variants by introducing specific mutations to the chosen residues (**Table 1 and S1 Table**). Residues belonging to the first and second shell of pkDAAO were mutated to alanine (variants 1–16 in **Table 1**) and, additionally Y55 was mutated to all other 18 residues (variants 16–34). Finally, (alanine or glycine) mutations and deletions were introduced in the active site lid loop and combined with Y55A mutations, both for pkDAAO and hDAAO (variants 37–46).

**Table 1:** List of DAAO variants designed for screening/measurement of their biochemical activity towards the substrates: 19 D-amino acids and glycine as well as a few homologues.

S. No	Origin	Location	Modification	Experiment
1–15	pkDAAO	First and second shell	Alanine mutations of L51, Q53, P54, Y55, T56, N96, N134, I215, H217, Y224, Y228, I230, F242, R283, G313.	Pilot screening
16–34	pkDAAO	Y55	Wild type Y55 and its 18 amino acid substitutions (except alanine)	Pilot screening and Measurement
35–36	pkDAAO	Active site ‘lid’ loop residues 223–225 I223-Y224-N235), and Y55	Glycine mutations to lid loop residues: I223G-Y224G-N235G, and their combination with the Y55A mutant: Y55A- I223G-Y224G-N235G	Pilot screening
37–41	pkDAAO	Active site ‘lid’ loop residues I223-Y224, and Y55	Alanine mutations of I223 and Y224, and their combinations with Y55A mutation: I223A, Y55A-I223A, Y55A-Y224A, I223A-Y224A and Y55A/I223A/Y224A	Pilot screening
42–46	pkDAAO and hDAAO	Active site ‘lid’ loop deletion, and Y55	‘Lid’ loop residues R221 and N225 joined (by deleting G222-I223-Y224 residues), in combination with their corresponding wild types and Y55A mutations	Pilot screening and Measurement
47–52	pkDAAO and hDAAO	Y55 and T56 or L56	Swap mutations interchanging T56L/L56T positions between pkDAAO and hDAAO in combination with Y55A or Y55W mutations	Measurement

### High-throughput screening of the engineered DAAO variants

An available high-throughput screening method [46] was employed to screen the biochemical activity of DAAO variants for their activity towards 19 D-amino acids and glycine (as well as substrate analogues) to clarify the role of specific residues. Commercially available pkDAAO, wild-type DAAO and wild-type glycine oxidase constructs, as well as empty expression constructs, were included as controls. The screening results revealed that most alanine variants (1–16 in **Table 1**) lost their activity (**S1 Fig**). However, among the alanine mutants, the Y55A mutant was active towards many substrates, while T56A, N134A, Y228A and G313A mutants displayed a comparable activity towards the high-affinity D-Met, D-Phe, D-Pro and D-Tyr substrates. Moreover, the Y55A mutant presented a different specificity towards most of the D-amino acid analogue substrates when compared to the wild-type pkDAAO (**S2 Fig**). For instance, the Y55A activity against D-homoserine and D-hydroxyproline decreases in comparison to wild-type pkDAAO. Such a result confirmed the relevance of the Y55 position. The pkDAAO T56A mutant displayed some resilience to mutation by retaining activity and also improving substrate specificity.

The variants 35–36 in **Table 1**that contained glycine mutations to the active site lid loop residues were then screened along with the wild-type and Y55A variants of pkDAAO (variants 16 and 4 in **Table 1**). The effect of glycine mutations to the lid loop residues in combination with the Y55A mutation was characterized to assess the change in solvent access through the lid loop residues due to the loss of their side chains. When compared to the variants with glycine mutations, that displayed a loss of activity towards most substrates, Y55A retained or raised its activity towards most substrates (**S3 Fig**). Additionally, to investigate the effect of each of the side chain of residues (222, 223, and 234) of the lid loop in combination with the Y55 against their wild-type on pkDAAO activity, the variants with alanine mutations to Y55 and the lid loop residues (variants 4, 10, 16 and 37–41 in **Table 1**) were screened (**S4 Fig**). The Y55A mutant was again found to retain its activity towards most substrates, while the Y224A displayed a deficient activity towards the D-Ala and D-Val substrates when compared to their wild-type. Therefore, comparing either the glycine mutations of the lid loop residues or their individual alanine mutations, Y55A mutant presented a better substrate specificity profile when compared to the pkDAAO wild type. Loop deletion variants (residues 221–225 joined by removing the G222-I223-Y224 residues) in combination with Y55A or their corresponding wild types were subjected to further analysis to compare the influence of solvent access through the lid loop spanning the active site and Y55 in pkDAAO and hDAAO.

Finally, the lid loop-deletion variants of pkDAAO and hDAAO along with their corresponding wild types and their Y55A mutants (16, 4, 42–46 in **Table 1**) were screened. Variants with loop-deletion displayed deficient activity or inactivity towards most substrates (**S5 Fig**). Such a loss of activity identified in pkDAAO and hDAAO variants with mutation/deletion to their lid loop residues confirmed their crucial role [14,15] in securing the hydrophobic environment around the FAD, which is indispensable for the DAAO activity and substrate specificity. The Y55A variant again presented a slightly improved activity towards a few substrates when compared to its respective wild types.

The overall screening results of the selected pkDAAO and hDAAO variants suggested that (i) the Y55 and T/L56 could withstand mutations and maintain activity, (ii) the Y55 and T/L56 modulate substrate specificity, and (iii) the role of the lid loop residues (including Y224) is imperative in governing DAAO activity.

### Computational analysis of mammalian DAAO dynamics and solvent access

The selected mutations to Y55 and T/L56 of pkDAAO and hDAAO were located in the region regulating access to their active sites. However, investigation of substrate entry or products egress is distinctive for a particular substrate/product pair that remains difficult to interpret in a broader context [47]. Therefore, we decided to use water molecules as a kind of non-specific probe and performed detailed computational simulations to investigate possible mechanisms regulating solvent access to the active site to identify changes introduced by specific mutations. Molecular Dynamics simulations (MD) of DAAOs in water were done using AMBER package [48], the tunnel network analysis was performed using CAVER [49], and a water tracking study was done using AQUA-DUCT software [50]. The mentioned procedure was applied for pkDAAO, hDAAO and selected hDAAO mutants. Additionally, accelerated Molecular Dynamics simulations (aMD) of hDAAO Y55A/L56T with indol-3-pyruvate (IND), which is the reaction product of D-Trp substrate, was studied using AMBER package [48]. (see ‘Materials and Methods’ section for details).

### Comparison of tunnel networks, solvent access pathways and crucial residues in pkDAAO and hDAAO

**Fig 1:**
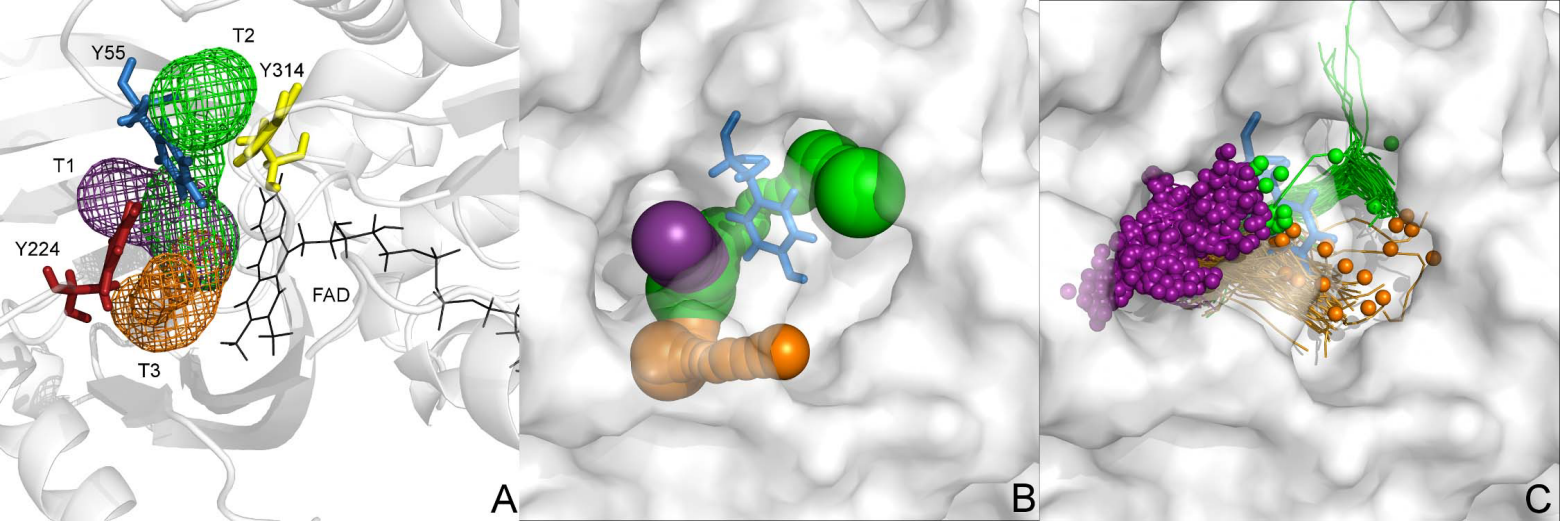
Geometry of main entities allowing entry to the active site of hDAAO. (A) tunnels represented by mesh, residues dividing tunnels by sticks, protein by cartoon, cofactor FAD is shown in black (wireframe), (B) (protein rotated approximately 30°), tunnels identified in single frame, tunnels represented by balls, protein by the solvent accessible surface. (C) tunnels and inlets of water molecules identified in entire MD simulation, tunnels represented by lines, water inlets by small spheres, protein by the solvent accessible surface. Tunnels calculated by CAVER, water inlets by AQUA-DUCT, picture prepared by PyMOL.

MD simulations combined with CAVER analysis were run to search for tunnels that could provide access to the pkDAAO and hDAAO active sites. Three tunnels, T1, T2 and T3, whose entrances/exits were located in the vicinity of the lid loop, were identified as the main access pathways. Other tunnels that were shown to be very narrow and long, or identified rarely, were discarded from further analysis. Y55 from the 53–57 loop separated the T1 exit from the T2 exit, while Y224 from the lid loop separated the exits of T1 and T3 (**Fig 1)**.

The geometry of the tunnels of both pkDAAO and hDAAO were quite similar. However access to the pkDAAO active site remained hindered (**S2 Table, S6 Fig**). The tunnel-opening events of pkDAAO were detected less frequently, were narrower and also of shorter duration than in hDAAO. T1 was the predominant tunnel in hDAAO. T2 and T3 were identified less frequently and were also narrower. However, the T2 and T3 tunnels opened remarkably during MD simulations. Since CAVER analysis does not provide information about the role of specific tunnels in ligands transportation, we decided to track all water molecules entering the active site during simulations using AQUA-DUCT software. It provided information about identified inlets and outlets of solvent molecules accessing the space constituted by the aromatic network of the Y55, Y224 and Y314 (**Fig 1C**). These inlets and outlets correspond to the exits of the T1, T2 and T3 in pkDAAO and hDAAO and further confirm the differences between the analysed proteins (**S6 Fig**). The total number of water molecules penetrating the pkDAAO active site was fewer than that seen with the hDAAO active site (583 vs 644) and more importantly shows different geometrical distribution (**Table 2, S6 Fig**). In pkDAAO two tunnels T1 and T3 are widely used for water access to the active site, whereas in hDAAO, this role is predominantly fulfilled by the T1 tunnel.

**Table 2:** Number of water molecules entering and leaving active site by main tunnels. **Data calculated from AQUA-DUCT results from 4 independent 50 ns long simulations**.

	pkDAAO inlets	hDAAO inlets	hDAAO Y55A inlets	hDAAO Y55A/L56T inlets
T1	379 ± 470	613 ± 106	1050 ± 380	1367 ± 442
T2	22 ± 24	8 ± 6	21 ± 17	701 ± 683
T3	257 ± 147	23 ± 23	62 ± 106	23 ± 37
SUM	583 ± 363	644 ± 111	1133 ± 355	2091 ± 627

**Fig 2:**
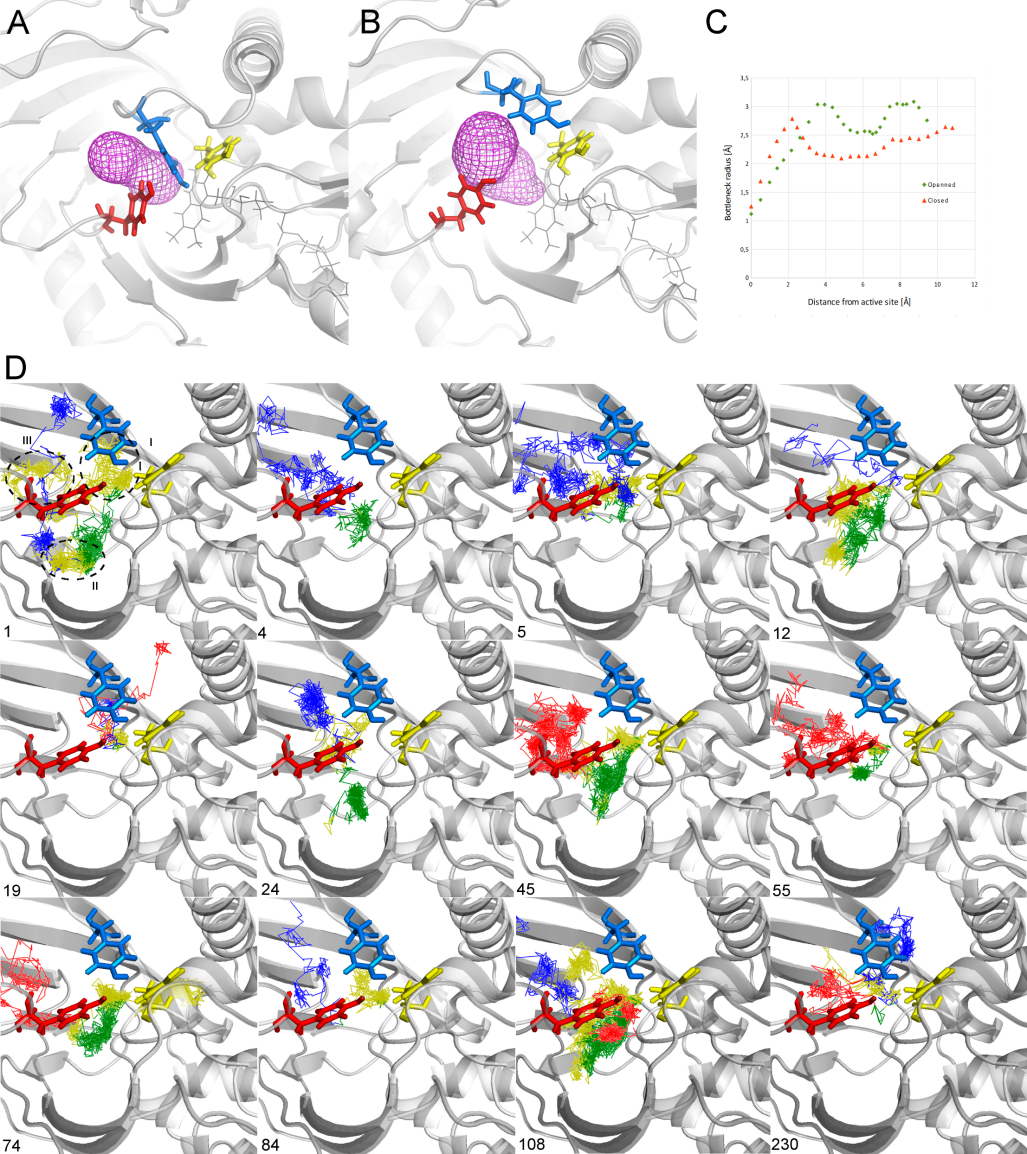
Geometry of tyrosine’s controlling access to the main entrance of the active site of hDAAO. (A) closed conformation (A—at 2452ps of MD simulations) and opened one (B—at 36838ps) shows the movement of Y55 side chain and shape of the main tunnel calculated by CAVER. The calculated profile of tunnels (C) shows significant narrowing of the tunnel when Y55 rotates and is sandwiched between Y224 and Y314. The Y55 is probably responsible for water molecules trapping inside active site cavity, (D) trajectories of selected water molecules which has entered active site during MD simulations calculated by AQUA-DUCT. Each figure presents trajectory of single water molecule divided into 4 parts: inside active site cavity (green line), from surface to the active site cavity (red lines), from active site cavity to the surface (blue lines) and that parts when water has left active site and travel back again due to geometrical constrains (yellow line), the number corresponds to raw path numbering by AQUA-DUCT. Please note that most of yellow part is located below Y55 residue (region I, visible on path no. 1, 5, 12, 19, 24, 45, 74, 84, 108, 230) or below Y224 residue (region II, visible on path no.1, 12, 74, 108). Only in two examples, water was traveling smoothly through analysed region (4,55) and in two cases, the water was trapped between Y224 and Y55 and had to re-enter the active site prior to release (region III, visible on path no.1 and 108). For clarity of pictures, regions are shown on the first trajectory only. Pictures visualised in PyMOL.

The results from the combined CAVER and AQUA-DUCT analysis suggested that the Y55 residue, along with Y224 and Y314, controls the properties of T1, T2 and T3 tunnels. The Y55 residue anchors the closed conformation of Y224 and the lid loop, to enable the hydrophobic environment required for the reaction. Meanwhile, Y224’s conformational flexibility is favourably assisted by interactions with Y314 from the adjacent loop. The closed orientation of the Y55 aromatic side chain is sandwiched between Y224 and Y314 and probably is responsible for water molecules trapping inside active site cavity (**Fig 2).** Comparison of the trajectories of water molecules either entering or leaving the active site shows that only in few cases water was not trapped (water path no. 4 and 55 on Fig 2D). In most cases, water molecules had to wait prior to release in a region in the vicinity of Y55 or Y224 side chain (yellow part of trajectories on Fig 2D). By taking the conformational plasticity of Y224 and the lid loop into account, we identified that the tight anchoring guaranteed by Y55 plays a major role in influencing access to the active site.

### Access modulation in hDAAO variants with Y55A mutation

**Fig 3:**
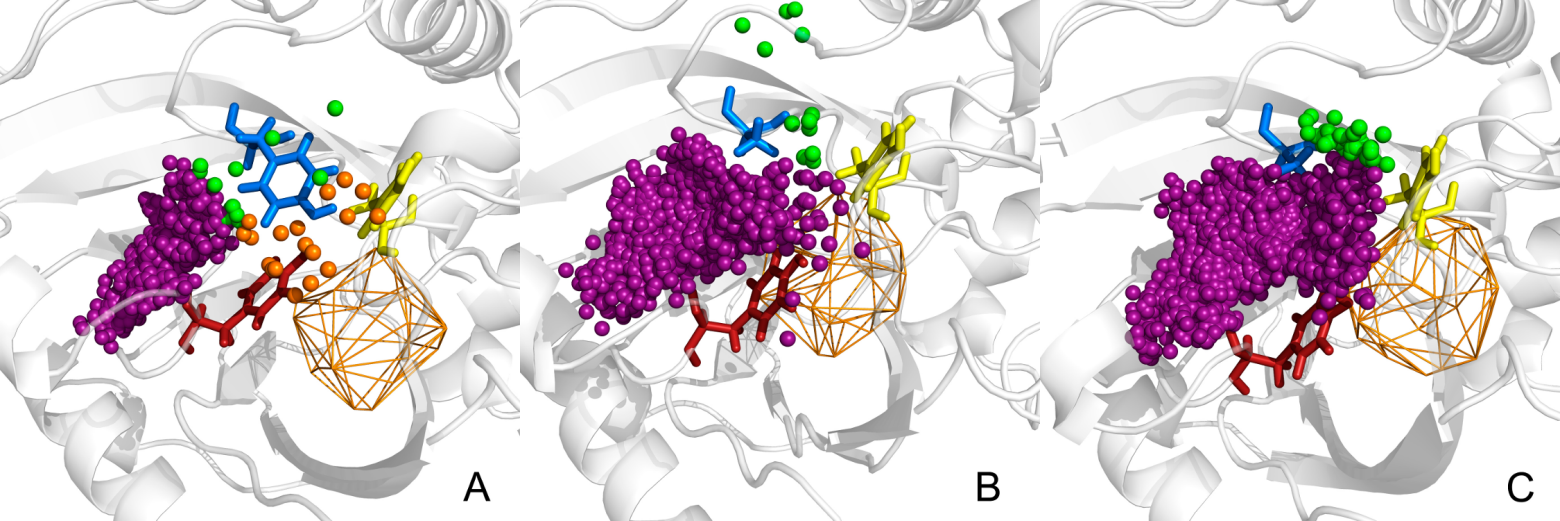
An examples of AQUA-DUCT results of tracking of water molecule passing during 50 ns of MD simulations through active site of: (A) hDAAO, (B) Y55A hDAAO, (C) Y55AL56T hDAAO. Protein shown as cartoon, active site object as orange wireframe, Y224, Y55 and Y314 as red, blue and yellow sticks, respectively. The inlets of water molecules entering/leaving the protein scope shown as small spheres.

**Fig 4:**
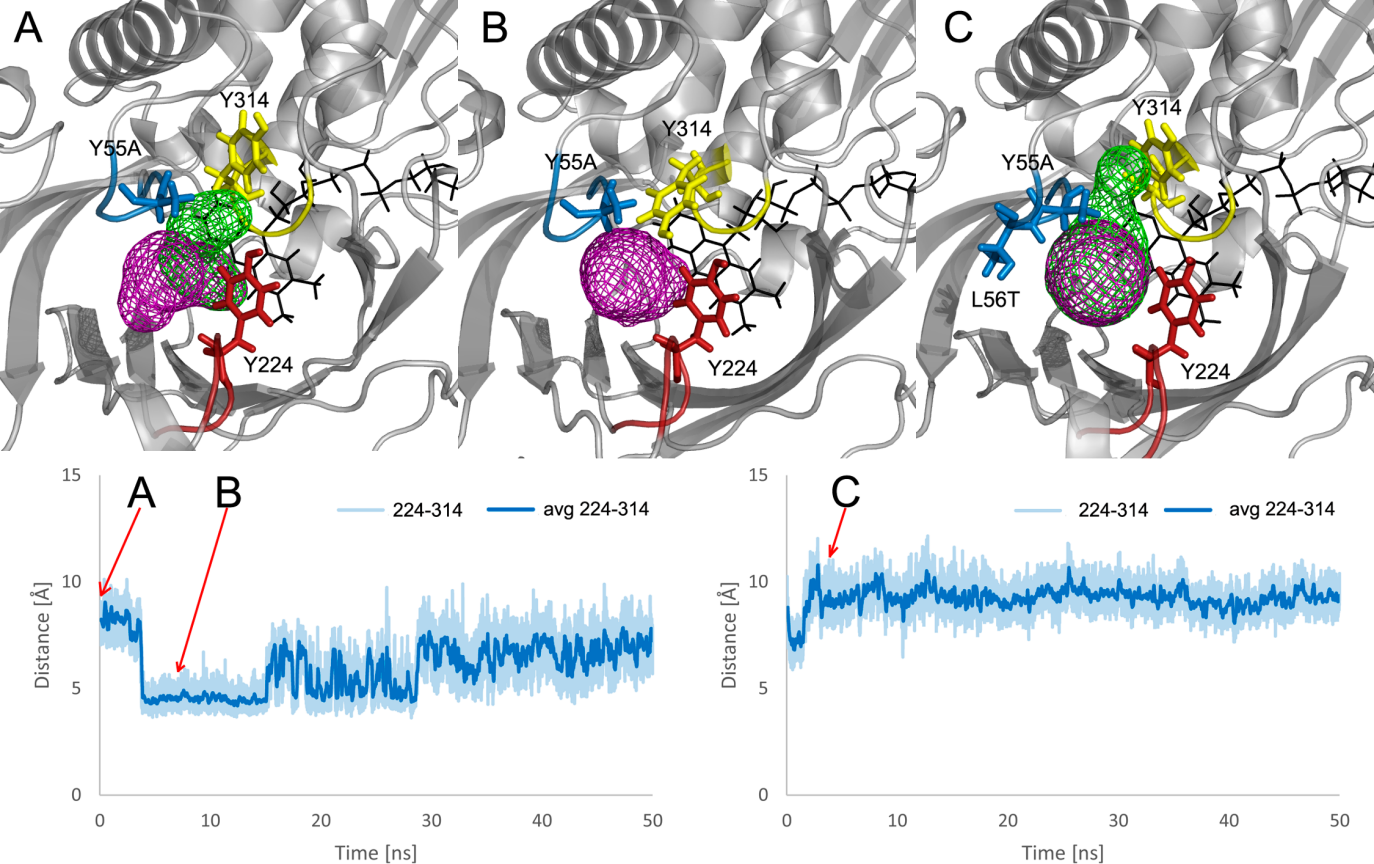
Dynamics of key residues in hDAAO Y55A and Y55A-L56T mutants during 50 ns MD simulations: Structures corresponding to particular snapshots (upper panel), the distance between Y224 and Y314 residues (lower panel). (A) The open conformation of Y314 in Y55A mutant, (B) closed conformation of Y314 in Y55A mutant, and (C) open conformation of Y314 in the Y55A-L56T mutant.

We introduced *in silico* an alanine residue in the place of Y55 residue in hDAAO using FoldX [51]. Only the most stable Y55A conformation was retained. Despite the loss of the large tyrosine side chain (which could rotate significantly), the main tunnel parameters of the Y55A mutant remained similar to the wild-type (**S2 Table**). However, the Y55A mutation dramatically impacted solvent access to the active site. The single large surface of water inlets suggests the apparent vanishing of the borders between the T1, T2 and T3 tunnels’ exits. The mutation greatly facilitated water access, as 1133 ± 355 water molecules were found to penetrate the active site of the hDAAO Y55A mutant in comparison to the 644 ± 111 found in the wild type (**Table 2, Fig 3A and 3B**). Also, the average time for a single water molecule to travel through particular tunnels, as well as an average time the water molecule to spend in the active site cavity is the longest for pkDAAO, and shortest in the designed mutants (**Table 3**). Close inspection of the surroundings of the tunnels’ exits indicated that Y314 rotated and anchored the lid loop and Y224 is mimicking Y55’s behaviour in the wild type (**Fig 4A and 4B**). The lack of the bulky aromatic side-chain of the tyrosine in the Y55A mutant made the space for the Y314 side chain rotation.

**Table 3:** Average time of single water molecule trajectory along analysed tunnels (T1, T2 and T3) and average time of water molecule stay in the active site cavity (AS). **Data calculated from AQUA-DUCT results from 4 independent 50 ns long simulations**.

	pkDAAO Time [ns]	hDAAO Time [ns]	hDAAO Y55A Time [ns]	hDAAO Y55A/L56T Time [ns]
T1	5.10 ± 3.13	3.17 ± 0.63	1.24 ± 0.19	1.27 ± 0.52
T2	4.70 ± 1.22	2.36 ± 1.05	1.26 ± 0.50	1.09 ± 0.47
T3	5.79 ± 3.53	2.54 ± 0.58	1.01 ± 0.29	2.45 ± 1.22
AS	4.04 ± 2.68	2.57 ± 0.64	1.01 ± 0.16	0.99 ± 0.52

We further studied the effect of the gain/loss of the (–OH) hydroxyl group in pkDAAO and hDAAO through T56L and L56T mutations respectively. An L56T mutation was *in silico* introduced in the hDAAO Y55A. Marked changes in the access pathways of hDAAO double mutant Y55A-L56T (**S2 Table**) came to the fore. Key differences included the increase of the bottlenecks of all tunnels. Active site accessibility markedly improved, with over 2000 water molecules passing through the active site (**Table 2, Fig 3C**). The dynamics of the loop containing residues 53–57, together with the lid loop (especially the A55-Y224 distance), chiefly controlled access and the bottleneck radii of tunnels, similarly to the hDAAO wild-type and Y55A single mutant. The additional L56T mutation appeared to stabilize an open conformation between the side chains of the Y224 and Y314 residues. The observed modulation of lid loop dynamics suggested potential facilitation of large substrate/product transportation (**Fig 4C).**

### Egress of reaction products

**Fig 5:**
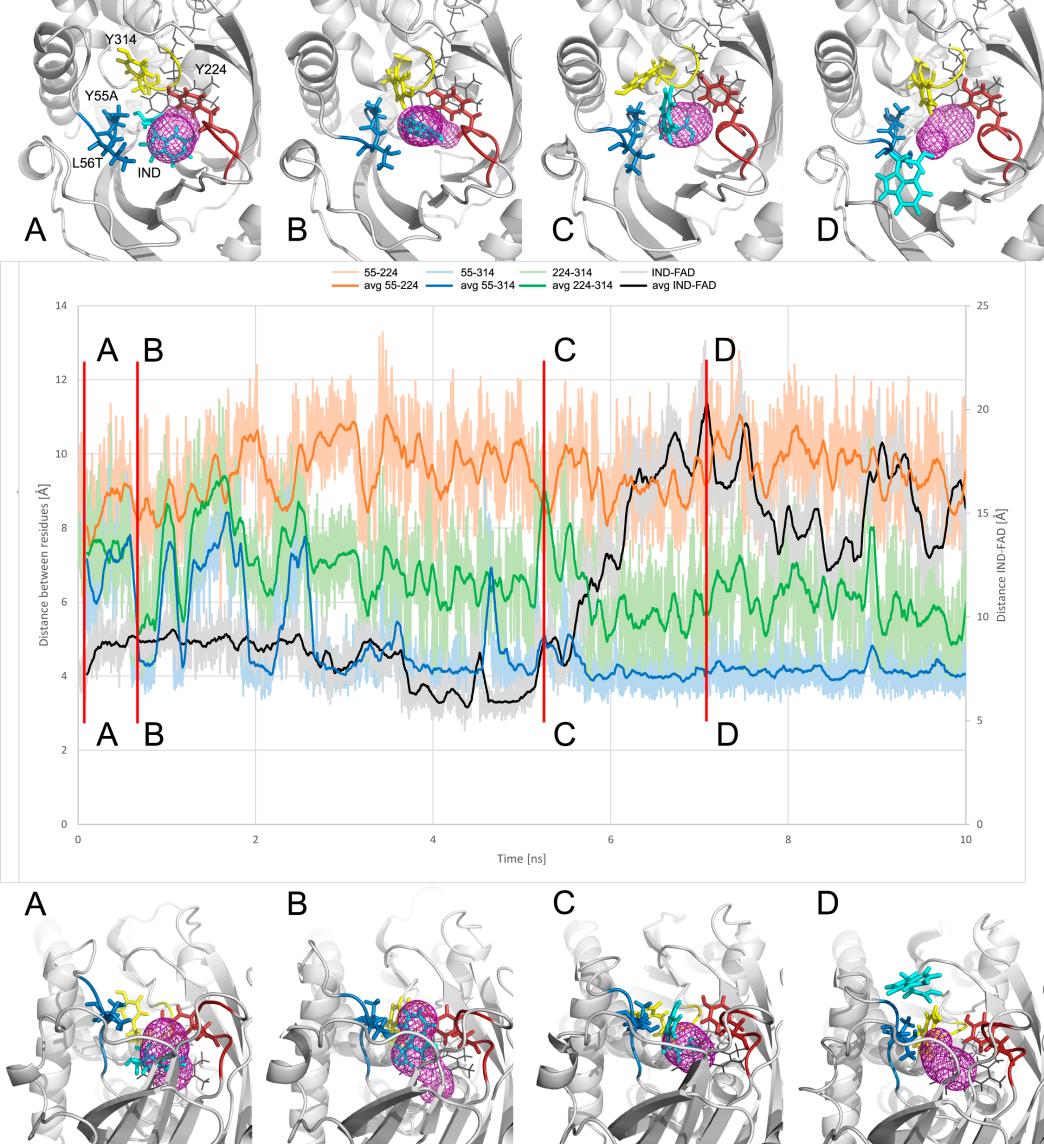
Passage of D-Trp product (IND: Indol-3-pyruvate in cyan stick representation) in hDAAO Y55A-L56T double mutant. Distances between key residues (A55-Y224-Y314) with respect to the distance between IND and FAD cofactor (middle panel); geometry of active site entrance corresponding to chosen snapshot (lower and upper panel; panels rotated by approximately 90^o^).

Egress of indol-3-pyruvate (IND) which is the reaction product of hDAAO’s activity towards its largest substrate, D-Trp, was studied. Our analysis revealed fast IND egresses from the hDAAO Y55A/L56T double mutant active site. The analysis of egress pathways and the corresponding conformational changes shed light on the orchestrated tunnel-opening events and the increased Y224-Y314 distance, which enabled IND egress. The lack of the large aromatic side chain of Y55, which was previously anchoring the Y224 from the lid loop in the wild type, facilitated the wide opening required for product egress **(Fig 5)**.

### Influence of substrate binding for active site accessibility

Our simulations suggest that the proposed combination of mutations (Y55A + L56T) greatly facilitates the access of water to the sites of action and the hydrophobic secondary binding pocket. However, such facilitation would be in contrast with the previous analyses [1,3,6,8–10] that underlined a hydrophobic environment around FAD being imperative for the hydride transfer step of substrate oxidation. Therefore, we investigated further how the binding of the substrate would modify the tunnel network. The D-Trp substrate was docked into the active site of hDAAO Y55A-L56T double mutant. 50 ns MD simulations showed that the D-Trp substrate in the active site decreased the site’s accessibility, as expected (**S2 Table**). The tunnels were less frequently detected, even less than in the wild type of pkDAAO, the most closed structure investigated so far, and was much narrower than in the mutant without reaction substrate.

### Biochemical analysis of pkDAAO and hDAAO selected variants

Variants containing mutations to Y224 or the lid loop showed a marked activity decrease and were discarded from further biochemical activity measurements. Only variants containing specific mutations of the Y55 and T/L56 residues (and their combinations) were further characterized: variants 4, 16–34 and 47–52 (**Table 1**). We proceeded with large-scale bacterial expression of the variants and subsequently purified them to homogeneity. Oxygen consumption measurements were used to calculate specific activity (**S3 Table**).

**Fig 6:**
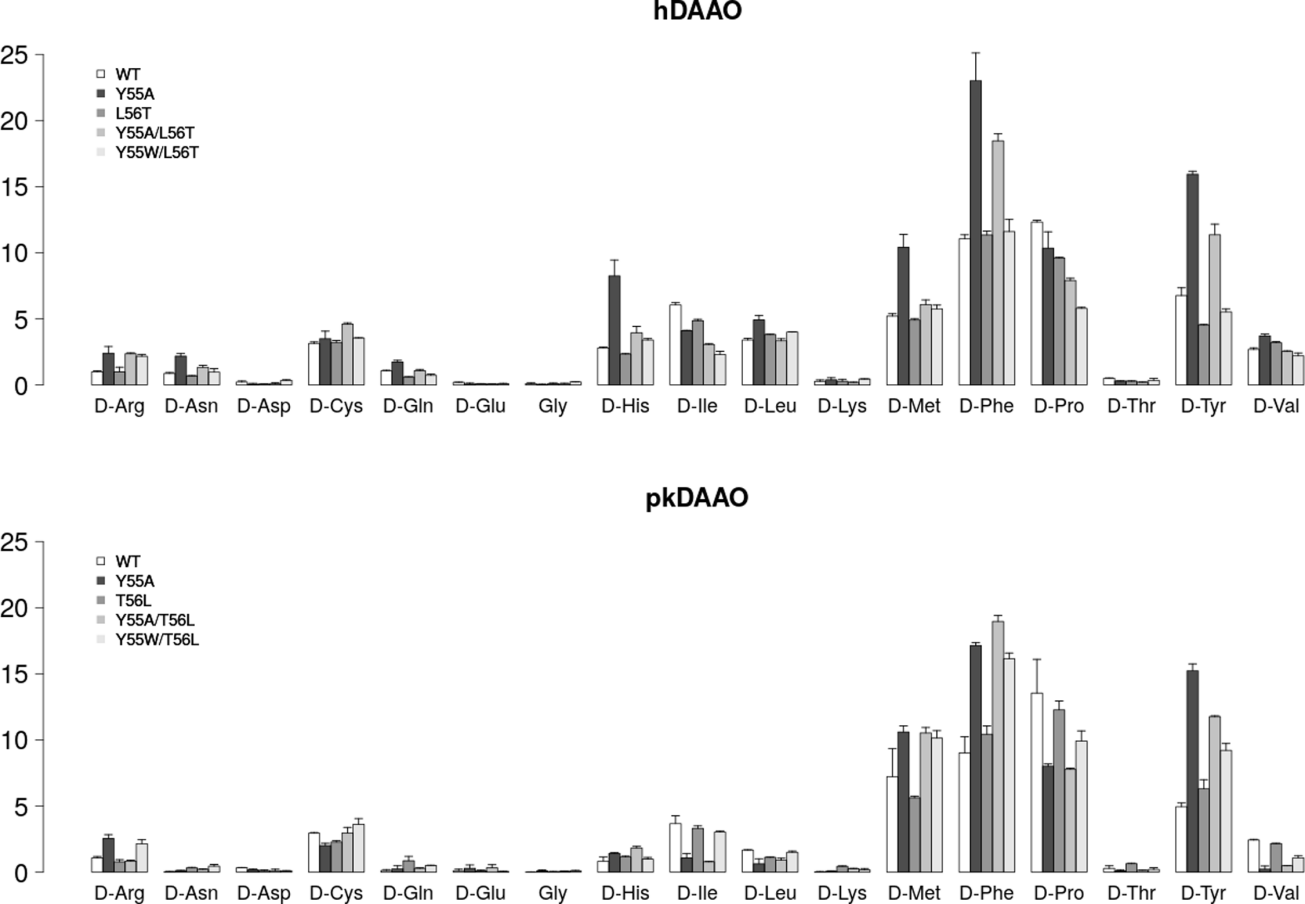
Enzyme specific activities (µmol/min/mg) of pkDAAO and hDAAO variants (all measurements in two replicates, from S3 Table over 16 D-amino acids and glycine substrates.

The overall behaviour of both the pkDAAO and hDAAO variants towards most substrates were similar (**Fig 6**). Nevertheless, hDAAO variants containing same mutations (such as Y55A and Y55P, or T56A/L56A) resulted in higher specific activity when compared to their corresponding pkDAAO variants.

Principal component analysis (PCA) of the specific activity of pkDAAO and hDAAO variants (**S7 Fig)** shows separation in the variants based on their origin (pkDAAO and hDAAO). However, wild-type pkDAAO and hDAAO and their T56L or L56T mutations, respectively appear in close proximity in PCA space, reflecting the similarity in their functional properties. The difference in behaviour of pkDAAO Y55P is driven by its higher activity (see **S3 Table**) towards D-His, D-Met, D-Phe, D-Trp and D-Tyr.

**Fig 7:**
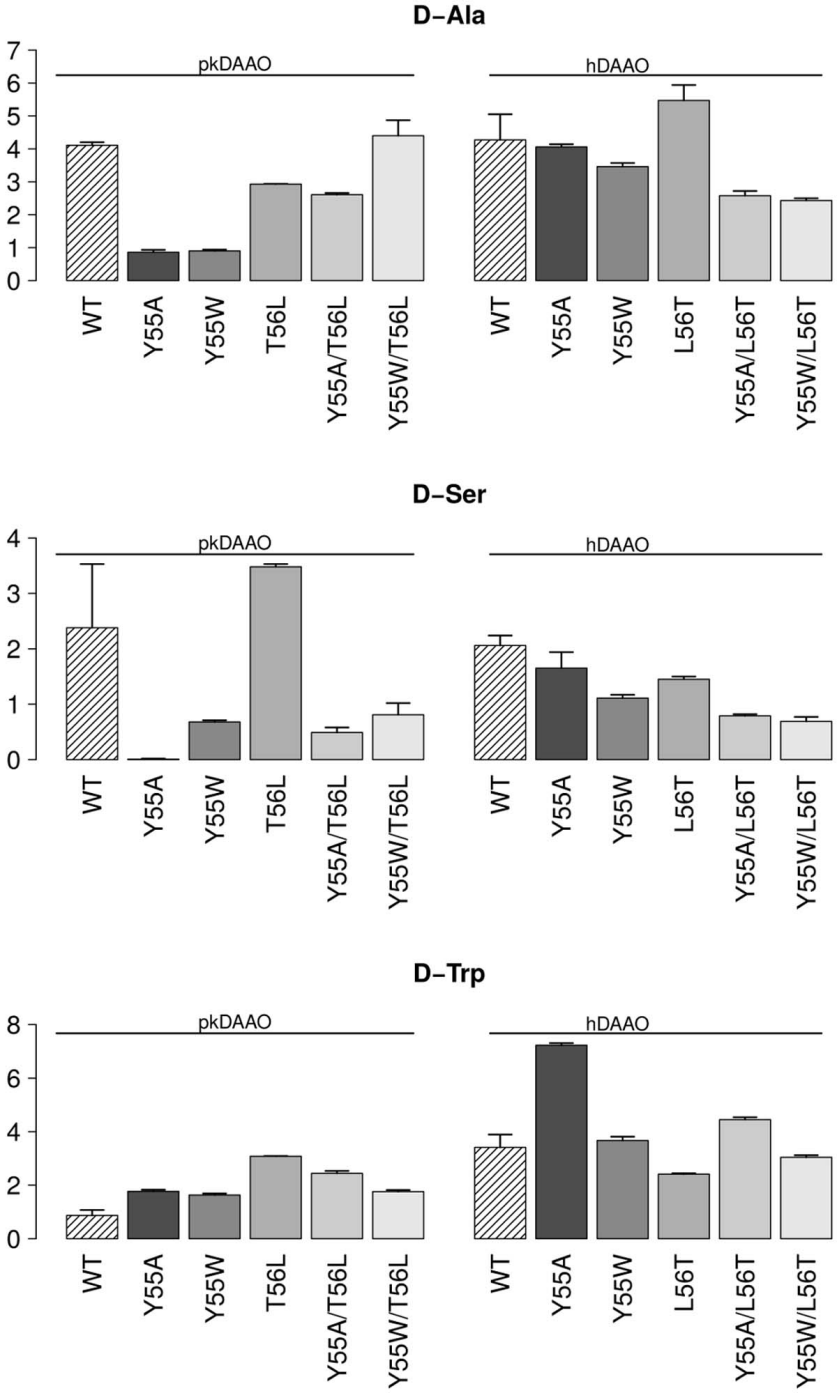
Specific activity (µmol/min/mg, from S3 Table) of pkDAAO and hDAAO towards the substrates: A) D-Ala, (B) D-Ser, and (C) D-Trp.

Moreover, we focused our attention towards the activity pattern of pkDAAO and hDAAO variants (**Fig 7A, 7B and 7C**) regarding the clinically relevant substrates [29–33,37]. We found no variation of specific activity, and at times only a mild variation, of the variants with mutations at position 55 and 56 towards D-Ala and D-Ser substrates, but a notably increased specific activity towards D-Trp in the case of hDAAO Y55A. In particular, hDAAO Y55A exhibited almost twice the activity towards the D-Trp substrate in comparison to the other hDAAO variants (**Fig 7C**).

## Discussion

Our focus was to identify residues in the first and second shell of the active site and lid loop of mammalian DAAOs that could help modulating activity/substrate specificity through facilitated product egress. The lid loop (Y224 residue, in particular) of the mammalian DAAOs is considered to be primarily responsible for their (slower) product release and probably for substrate specificity variations [1–3,5,6,9,12–19]. Residues from other loops (such as 43–56 loop consisting Y55 and T/L56) that are around the mammalian DAAO active sites were so far neglected for a detailed analysis. In contrast to the decreased activity that we identified in our analysis due to either removal or glycine/alanine mutations to the residues of the mammalian lid loop, Y55 sustained mutations and improved enzyme characteristics. In the yeast DAAOs such as TvDAAO and RgDAAO [20–23], no loop similar to the mammalian lid loop that influences enzyme properties had so far been identified. Therefore, we used structure-based multiple sequence alignments of yeast and mammalian DAAOs (data not shown) to identify that RgDAAO F58 and TvDAAO F54 are the mammalian DAAO Y55 position equivalents [20,22], which are located in loops around their binding pockets. The alignment also revealed that the residue at position 56 is threonine for pkDAAO, but leucine for all other mammalian DAAOs, including hDAAO. The T56/L56 difference between pkDAAO and hDAAO remained unexplored. Moreover, the conclusion of pkDAAO engineering study [24] through Y55F mutation suggested the loss of hydroxyl group (-OH) at the sidechain is inconsequential for activity.

In our research, we took advantage of the growing awareness of the relevance of solvent flow, transport pathways and gating residue dynamics regulating enzyme properties [40–42]. We used a combination of experimental and computational approaches to fine-tune mammalian DAAO properties. We combined all the clues on structural and structure-based computational [1–3,5–7,9,10,12–19,44] as well as the functional relevance of DAAO residues with our biochemical and computational analysis. The synchronised dynamics of a network of aromatic residues, Y55, Y224, Y228 and Y314, from the adjacent active site-spanning loops of mammalian DAAOs controls access to the active site. Such synchronisation of the residues and loop dynamics correlates to the opening and closing of tunnels, to the regulation of product release and probably the substrate entry. When Y224 and the lid loop were in open conformation, the hydrogen bonding that existed between Y228 and the ligand/inhibitor (similar to that between the ligand/inhibitor and both R283 and G313) was lost. The Y55 residue containing the (–OH) hydroxyl functional group compensated for that through additional hydrogen bonding to the ligand/inhibitor in the hydrophobic secondary binding pocket in such ligand-bound structures [16–19]. Notwithstanding the hydrophobicity of the secondary binding pocket of mammalian DAAOs, a few water molecules were located in its vicinity. The probable effects of the polar side chain of T56 pkDAAO or the hydrophobic side chain in L56 hDAAO lining the secondary binding pocket on the availability of water molecules in the vicinity, as well as the flow of solvent in/out of the active site, remained unexplored.

In our research, we have focussed our attention on the role of residue Y55 and the regulation of solvent passage around the region controlled by the lid loop. By combining all these aspects, we could realize (i) the relevance of lid loop of mammalian DAAOs, (ii) the importance of the residues on the loops located at the funnel-shaped entrance of mammalian DAAO active-site and secondary binding site and (iii) the predominant role of Y55 (and Y314) in solvent access-substrate/inhibitor positioning in the active site/secondary binding site when Y224 sidechain assumes alternate (open/closed) conformations.

Our analysis highlights the crucial roles of Y55 residue in pkDAAO and hDAAO. The Y55 residue (i) anchors the lid loop in the closed conformation, (ii) separates the exits of the T1 and T2 tunnels, while the dynamics of Y314 locks the dynamics of Y55, (iii) modifies tunnel properties, and (iv) regulates solvent access, water trapping inside active site cavity and product release. The results of the MD simulations suggest that in those hDAAO variants involving the Y55A mutation, Y314 takes over a similar anchoring role to that carried out by Y55 in the wild type. The lack of bulky Y55 side chain makes space for Y314 rotation. Such a situation provided an unexpected stabilisation of the closed conformation of the lid loop (and Y224) by Y314 when compared to Y55 in the wild-type hDAAO. Although the anchoring residues were successfully substituted, the modified properties of the gate-anchoring residue pair changed that significantly improved access to the active site, which can be seen from water access analysis. Such results explain the improvement identified in the activity of the Y55A mutant towards bulky substrates, such as D-Trp, in the high-throughput screening results.

However, the larger Y224-Y314 distances in those hDAAO variants containing the Y55A mutation suggest the facilitation of both the opening of the lid loop and access to the active site. This phenomenon became apparent by the remarkable increase in the exit/entry points for the solvent molecules as identified by our AQUA-DUCT analysis. This effect can be further improved by the introduction of additional L56T mutation. Our extended aMD simulations suggested that the Y314 side chain rotation controls the process of product egress in the Y55A/L56T mutant. The improved accessibility of the active site, suggests easier access of bulky substrates but also water molecules into catalytic site. The docking results suggest that, despite the increased access of the solvent to the active site due to Y55A mutation, loops surrounding the active and rear side cavity undergo sufficient conformational change after substrate binding to protect the hydrophobicity around FAD, which is required for the first step of the reaction.

The dynamics of the bulky aromatic side chain of the Y314 residue of pkDAAO and hDAAO also shields the required hydrophobic environment of G313 and T316 from the surrounding solvent. Any further modification of Y314 should be performed carefully, since G313 and T316 in the neighbourhood are involved in crucial (hydrogen bonding) interactions to either the substrate/product [2,3,5,29] or the FAD cofactor. Therefore, we recommend Y314 for future extensive computational and engineering analysis, by mutating it in combination with the Y55 and Y224 gates as well as the substrate-positioning Y228 and R283 residues.

Since the wild-type pkDAAO active site access remains more hindered in comparison to the wild-type hDAAO, we suspect that its Y55 substitutions ease access to a greater extent than those of hDAAO, which is reflected in their substrate specificity profiles (**S3 Table**). Results from the specific activity measurements further confirm the role of Y55 and T56/L56 in controlling access pathways and consequently enabling additional access that facilitates product egress, which is the rate-limiting step of the mammalian DAAO catalytic cycle. Hence, mutations of Y55 and T/L56 improved substrate specificity profile of mammalian DAAOs, including the clinically relevant D-Ser, D-Ala, and D-Trp substrates as well as substrate analogues. Such optimisation, as well as delicate modulation (such as hDAAO Y55A and Y55W substitutions in combination with L56/T56 swap-mutations) of substrate specificity, renders the identification of these variants containing non-obvious targets attractive for industrial and clinical applications of DAAO. We recommend our hDAAO variants for further investigations of inhibitor/ligand design and substrate specificity modulation since they complement the variations in hDAAO expression/activity/substrate specificity identified in severe diseases [2,5,24,27–31], especially schizophrenia [32,35]. All in all, we engineered DAAOs with a low mutation load and modulated their substrate specificity without losing activity, including that towards the clinically relevant substrates, such as D-Ala, D-Ser and D-Trp. Our rational approach showcases the advantages of predicting promising candidates that influence and regulate solvent access pathways for engineering enzymes with optimised functional properties. Subtle modulation of solvent access and consequently substrate specificity also brings variants valuable for applications in single-pot reactions, enantiospecific reactions and enzyme cascades.

In summary, we identified a delicate modulation of specific activity in all the pkDAAO and hDAAO variants that we designed, without losing activity. Our computational analysis deciphered the sophisticated mechanism involved in the product release and the regulation of solvent access pathways through the synchronised dynamics of the Y55, Y224 and Y314 residues from adjacent loops. Those pkDAAO variants with substitutions to the Y55 residue that varied substrate specificity to a greater extent, such as the Y55P would be attractive for industrial applications. Although we have added to the available knowledge on the critical residues influencing access pathway modulation to the active site and the hydrophobic secondary binding pocket, further investigations are warranted.

## Materials and methods

### Materials

All reagents used were of analytical grade. All D-amino acids and its analogues as well as glycine substrates, 3,3’-Diaminobenzidine tetrahydrochloride (DAB), Peroxidase from Horseradish (HRP), cofactor flavin adenine dinucleotide FAD, sodium benzoate and lysozyme were purchased from Sigma-Aldrich.

### Preparation of plasmid constructs and mutagenesis

Codon-optimized gene constructs as SacI-[GENE]-SalI-TAA-XhoI were synthesised and subcloned into the multiple cloning site of the pET-28a (+) vector via SacI/XhoI by GenScript for all variants. UniProt identifiers (sequences) for the yeast TvDAAO, pig pkDAAO, human hDAAO and glycine oxidase (GO) that were synthesised/used for biochemical activity analysis in this study are Q99042, P00371, P14920, and O31616, respectively. GenScript performed all mutations described. All constructs and mutants were verified by automated DNA sequencing. Every expression product contains extra amino acids arising out of the restriction sites of the cloning vector: 38 amino acid stretch (including a His-tag, which is highlighted and underlined in the sequence) attached to the N-terminal {LTLRRRYTMGSS**HHHHHH**SSGLVPRGSHMASMTGGQQMGRGSEFEL} and two additional amino acids (VD) at its C-terminal.

### Screening E. coli with expressed Pig-DAAO

E. coli BL21 (DE3) harboring the pET-28a (+) vector containing the DAAO gene were grown in LB (Lysogeny Broth) medium, supplemented with 50 µg/ml kanamycin, at 37°C with shaking up to an optical density of about 1.2–1.5 at 600 nm, and protein expression was induced by addition of isopropyl β-D-1-thiogalactopyranoside (IPTG) to a concentration of 0.01mM. Cultures were grown for a further 18–20 hours. Cells were harvested by centrifugation and re-suspended in a small volume of supernatant to achieve a 25-fold concentration. 2.5 µl volumes of these concentrates were dot blotted onto a nitrocellulose membrane (Protran BA85 from Whatman) and dried in the air. The membranes were incubated in 1% (w/v) BSA and 0.2% (v/v) Triton X-100 in 100mM KP_i_ (pH 7.0) for 20–30 minutes at RT and washed with 50 mM sodium pyrophosphate (pH 8.3). The E. coli cells were then activity stained by addition of 10 mM D-amino acid, 0.2 mg/ml 3,3’-Diaminobenzidine tetrahydrochloride (DAB), 0.5 mg/ml Peroxidase from Horseradish (HRP) in 50 mM sodium pyrophosphate (pH 8.3). Incubation was performed on a rocking platform, protected from (day) light, until the dots of E. coli cells with active DAAO/GO were visualised as brown spots.

### Protein expression and purification

Proteins (pkDAAO and hDAAO variants) were expressed in 800 ml cultures (Lysogeny Broth medium supplemented with 50 µg/ml kanamycin) of E. coli BL21 (DE3) transformed with the pET-28a (+) vector containing the DAAO/GO gene. Cultures were grown at 37°C with vigorous shaking up to an optical density of about 1.2–1.5 at 600 nm, and protein expression was induced by addition of isopropyl β-D-1-thiogalactopyranoside (IPTG) to a concentration of 0.01mM. Cultures were grown for a further 18–20 hours. Cells were harvested by centrifugation and stored frozen at -20˚C. Cell pellets were suspended up to 0.1 g/ml in 50 mM sodium pyrophosphate pH 8.3, 20 µM FAD and 1 mg/ml sodium benzoate. Lysozyme was added up to 1 mg/ml, together with a few crystals of DNaseI, and cells were lysed on ice for 1 hour. Cell disruption was subsequently completed by sonication on a Branson Sonifier B12 equipped with a 5mm tip, using four cycles of 30 s 40% output and 15 s intervals. Cell debris was precipitated by centrifugation. Ammonium sulphate was added to the supernatant to a final concentration of 150 mg/ml, and the solutions were stored O/N at 4˚C. The precipitate was collected by centrifugation and dissolved in His-Trap binding buffer containing 20 µM FAD and 1 mg/ml sodium benzoate. The DAAO in suspension was applied to a 1 ml HisTrap™FF column (GE Healthcare) and rinsed thoroughly with His-Trap binding buffer. DAAO was eluted with His-Trap Elution buffer and the yellow coloured fractions were pooled. DAAO was concentrated by a second ammonium sulfate precipitation (up to 200 mg/ml) at 4˚C, and the pellet was resuspended in a small volume of 50 mM sodium pyrophosphate pH 8.3, supplemented with 20 µM FAD and 1 mg/ml sodium benzoate, quickly frozen in liquid Nitrogen and stored at -80°C. The purity of the DAAO was checked using SDS-PAGE (Bio-Rad Mini-PROTEAN System) and PageBlue staining. Before use, DAAO isolate was desalted on an Econo-Pac^®^ 10DG Desalting Column (Bio-Rad) eluted with 50 mM sodium pyrophosphate pH 8.3. The UV-VIS spectrum in the range 250-600nm was measured on a Shimadzu UV-2501PC spectrophotometer. The concentration of every pkDAAO and hDAAO variant (**S3 Table**) was calculated using its molar extinction coefficient and average molar weight calculated from *http*:*//web*.*expasy*.*org/protparam/*. Finally, FAD was added up to a final concentration of 20 µM.

### Determination of specific activity

An OxyTherm Clark-type oxygen electrode system (Hansatech) was used to determine the initial oxygen consumption during the oxidative deamination of D-amino acid and glycine substrates. The assay solution (final volume, 1.0 ml) contained 10 mm D-amino acid or glycine substrate, 20 μm FAD, and about 10 μg enzyme in air-saturated 50 mM sodium pyrophosphate (pH 8.3) at 25°C. Data acquisition and analysis were performed using Oxygraph Plus software (Hansatech).

### Protein data bank accession codes

The structure factors and coordinates for the hDAAO and pkDAAO structures have been obtained from the Protein Data Bank [52] with accession codes 2DU8 and 1VE9, respectively. To save calculation time and computational costs, only the chain A was used in our study. We have assumed that the changes caused by mutations will have larger influence than that caused by dimerization. These assumption was justified by comparison of our data with previously reported [44,53].

### Protein structure preparation and classical molecular dynamics simulations

H++ server [54] was used to protonate each structure at pH 8.3. LEaP [48] was used to prepare protein structures to MD simulation using ff14SB force field [55], add counterions and immerse models in a truncated octahedral box of TIP3P water molecules and divalent ion parameterized for Particle Mesh Ewald and TIP3P water model. The FAD cofactor was parameterized based on work by Medvedev and Stuchebrukhov [56]. Amber 14 [48] was used to run multiple 50 ns long simulations for pkDAAO and hDAAO proteins separately. Minimization procedure consisted of 2000 steps, involving 1000 steepest descent steps followed by 1000 steps of conjugate gradient energy minimization, with decreasing constraints on the protein backbone (500, 250, 125, 75 and 25 kcal*mol^-1^*Å^-2^) and a final minimization with no constraints of conjugate gradient energy minimization. Gradual heating was performed from 0 K to 300 K over 25 ps using a Langevin thermostat with a temperature coupling constant of 1.0 ps in a constant volume periodic box. Equilibration and production were run using the constant pressure periodic boundary conditions for 2 ns with 1 fs time step and 50 ns with a 2 fs time step, respectively. The constant temperature was maintained using the weak-coupling algorithm for 50 ns of the production simulation time, with a temperature coupling constant of 1.0 ps. Long-range electrostatic interactions were modelled using the Particle Mesh Ewald method with a nonbonded cut-off of 10 Å and the SHAKE algorithm. The coordinates were saved at intervals of 1 ps. The trajectories were visually inspected with VMD (Visual Molecular Dynamics) program [57].

### Tunnels identification and analysis

The CAVER Analyst 1.0 [58] was used for preliminary tunnels identification and parameters adjustment. The standalone version of CAVER 3.02 [49] was utilised for the calculation of tunnels in the 50 ns simulations of the wild type and all mutants. For all systems 50000 snapshots from molecular dynamics production run were analysed. Water molecules or ligands were removed before the tunnel calculation, and the non-hydrogen atoms of the structure were approximated by 6 spheres of the size of a hydrogen atom. As initial starting points of the tunnel search the centres of gravity of atoms FAD N2, L51 CD2 and R283 CZ were selected. Starting points positions were automatically optimised to prevent its collision with protein atoms. Tunnels were calculated using a probe radius of 0.7 Å, shell depth– 3 Å, shell radius– 3 Å, clustering threshold– 5. Tunnels were clustered using hierarchical average-link clustering. CAVER 3.01 plugin for PyMOL [59] was used for visual inspections of calculated tunnels.

### Water tracking

AQUA-DUCT [50] software version 0.4.dev3 was used to trace water molecule paths that have entered or left the DAAO active site. In this version of AQUA-DUCT the paths are trimmed to fit exactly into the protein surface and also new method of clusterization was developed. AQUA-DUCT was tracking paths of all water molecules that were found within 5 Å of the N2 atom of the FAD cofactor. The scope was defined as an interior of the convex hull of C-alpha atoms of the protein. Each water molecule path was cut to fit the protein surface (Auto-Barber set to protein). All inlets were then clustered using Barber method with cutting correction to the van der Waals radius of the closest atom.

### Substrates/products docking

Docking simulations were performed with AutoDock Vina v1.1.2 software [60]. Ligands were initially processed by a set of standardisation procedures with the standardised tool software [61]. Starting 3D structures were generated by simple geometry optimisation by UFF [62] as implemented in RDKit [63]. Partial charges of Gasteiger-Marsilii type [64], selection of rotable bonds, and assignment of AutoDock Vina specific atom types were done automatically in AutoDock Tools suite v1.5.7 [65]. DAAO protein structure was obtained by averaging the first nanosecond (1000 frames) of production of “empty” double mutant Y55A-L56T. The protein structure was preprocessed using AutoDock Tools v1.5.7 suite. Amber partial charges were retained and AutoDock Vina atom types were assigned automatically. The size of the search space was set to 20 × 20 × 20 Å, and it was centered at expected binding site residues, i.e., at Y224, Y228, R283, A55, T56 residues. Docking simulations resulted in twenty different docking poses for each ligand. The maximal allowed energy range between the best and the worst binding mode was set to 3 kcal/mol. The global search exhaustiveness parameter controlling the search algorithm accuracy was set to 12. Final poses were manually selected after careful visual inspection.

### Accelerated MD simulations

Accelerated MD simulations were performed for human DAAO (an average structure from first nanosecond of cMD production run of A chain). The system consists of FAD cofactor and D-Trp product—indol-3-pyruvic acid docked in the active site of hDAAO. Product was parameterized using antechamber and parmchk from Amber 14 [48]. Minimization procedure consisted of 2000 steps, involving 1000 steepest descent steps followed by 1000 steps of conjugate gradient energy minimization, with decreasing constraints on the protein backbone (500, 250, 125, 75 and 25 kcal*mol^-1^*Å^-2^) and a final minimization with no constraints of conjugate gradient energy minimization. Gradual heating was performed from 0 K to 310 K over 25 ps using a Langevin thermostat with a temperature coupling constant of 1.0 ps in a constant volume periodic box. Equilibration was run using the constant pressure periodic boundary conditions for 2 ns with 1 fs time step. Production was run for 200 ns with 2 fs time step in constant pressure. The aMD implementation in AMBER [66] allows for the possibility of boosting the whole potential with an additional boost to the torsions (iamd = 3). alphaD is defined as the product of 0.2, 3.5 kcal/mol/residue, and the number of protein residues. EthreshD is defined as the product of 3.5 kcal/mol/residue and the number of protein residues (including FAD cofactor and product), added to the average dihedral based on average over 5000 steps of cMD production. alphaP is defined as the product of 0.2 and the total atoms. EthreshP is defined as the sum of alphaP and the average EPtot over 5000 steps of cMD production. The coordinates were saved at intervals of 2 ps. The trajectories were visually inspected with VMD (Visual Molecular Dynamics) program [57].

## Supporting information

S1 Table Variant list. (PDF)

S2 Table Main parameters of tunnels in pkDAAO wild type, hDAAO wild type, hDAAO Y55A, hDAAO double mutant Y55A/L56T. Freq–frequency of tunnels identified with probe radius 0.9 during 50 ns MD simulations; Avg B–average bottleneck radius [Å], Avg L–average length [Å].
(PDF)

S3 Table Enzyme specific activity (µmol/min/mg) of the purified pkDAAO and hDAAO variants measured (in two replicates) towards 19 D-amino acids and glycine substrates. Rows represent the DAAO variant, where P is pkDAAO and H is hDAAO. Columns represent the corresponding substrate used (D-Ala to D-Val).
(PDF)

S1 Fig Screening the activity of pkDAAO variants expressed in BL21 host cells: Equal amounts of crude cell lysates blotted on a membrane. The variants 1–15 from **S1 Table** were designed by mutating the first and second shell residues of pkDAAO to an alanine. Each panel in **S1 Fig** represents the activity of all the variants towards a specific substrate (D-Ala, D-Arg, A-Asn, to D-Val). Equal amounts of the BL21 host cell (that lack the expression vector, but similarly induced) crude lysates were blotted alongside to observe any background activity. The numbers mentioned beside the corresponding blotted samples (19–33) represent the following alanine mutants, where the variant numbers in **S1 Table** are shown within parenthesis. 19: L51A (1), 20: Q53A (2), 21: P54A (3), 22: Y55A (4), 23: T56A (5), 24: N96A (6), 25: N134A (7), 26: I215A (8), 27: H217A (9), 28: Y224A (10), 29: Y228A (11), 30: I230A (12), 31: F242A (13), 32: R283A (14), 33: G313A (15).
(TIF)

S2 Fig Screening the activity of pkDAAO wild type (Variant 16 in S1 Table) and Y55A (Variant 4 in S1 Table, which is 22 in S1 Fig) towards D-amino acids, glycine and D-amino acid analogues in crude cell lysates on the membrane (in duplicate). Equal amounts of the BL21 host cell (that lack the expression vector, but similarly induced) crude lysates were blotted alongside to observe any background activity. The columns (1–15) represent the following substrates. 1. Sarcosine (synonym: N-Methylglycine), 2. D-Norleucine, 3. D-Norvaline, 4. D-Ornithine monohydrochloride, 5. D-2-Aminobutyric acid, 6. D-Citrulline, 7. D-Homoserine, 8. D-Penicillamine (synonym: 3,3-Dimethyl-D-cysteine), 9. D−(−)-α-Phenylglycine (synonym: D-2-Phenylglycine), 10. cis-4-Hydroxy-D-proline (synonym: D-allo-Hydroxyproline), 11. D-Pipecolinic acid (synonym: D-Homoproline), 12. D-Methionine, 13. D-Arginine, 14. D-Serine, 15. D-Valine.
(TIF)

S3 Fig Screening the activity of pkDAAO variants (4, 16, 35 and 36 in S1 Table) on the membrane towards 19 D-amino acids and glycine substrates. Alongside the pkDAAO variants (approx. 50μM purified) also containing Y55A mutation or glycine mutation to the lid loop residues, in duplicate, an equal amount of the commercially available pkDAAO wild type (Calzyme) was blotted to compare activities. The rows represent each variant screened, where A is Y55A, GGG is I223G-Y224G-N235G and AGGG is Y55A- I223G-Y224G-N235G. Columns represent the substrate used to screen the activity of the variants, from D-Ala to D-Val.
(TIF)

S4 Fig Screening the activity of (Approx. 50μM purified) pkDAAO variants (4, 10, 16, 37–41 in S1 Table) on the membrane towards 19 D-amino acids and glycine substrates. Alongside the pkDAAO variants, in duplicate, an equal amount of the BL21 host cell (that lacks the expression vector, but similarly induced) was blotted to observe any background activity. The rows represent each variant screened, where the wild type, Y55-I223-Y224, is YIY, and the corresponding alanine mutation is denoted in red. Columns represent the substrate used to screen the activity of the variants, from D-Ala to D-Val.
(TIF)

S5 Fig Screening the activity of pkDAAO and hDAAO variants (4, 10, 16, 42–46 in S1 Table) containing loop deletion in combination with their corresponding Y55A and wild types. Crude cell lysates were blotted on the membrane, and the activities towards 19 D-amino acids and glycine substrates were analysed. Every panel in the figure represents the activity of the variants towards a specific substrate (D-Ala to D-Val), and BL21 host cells were dot-blotted as a control to compare background activity. Rows with either P or H denote the pkDAAO or hDAAO, respectively. In the columns, the following representation is followed: Y is the wild type, YΔ is the loop deletion mutant, A is the Tyr55Ala mutant, and AΔ is the double mutant involving both Tyr55Ala mutation and loop deletion.
(TIF)

S6 Fig Comparison of tunnels detected by CAVER (represented by lines) and water inlets (represented by small spheres) detected by AQUA-DUCT in pkDAAO wild type (A) and hDAAO wild type (B). Residues dividing tunnels are represented by stick (Y55 blue, Y224 red, Y314 yellow), protein by semitransparent cartoon), Panel on the right shows same region of the protein rotated approximately 90°.
(TIF)

S7 Fig PCA of the all DAAO variants whose specific activity (µmol/min/mg) is measured (from S3 Table). (TIF)

## Abbreviations

DAAO: D-amino acid oxidase
FAD: flavin adenine dinucleotide
PCA: principal component analysis
MD: molecular dynamics
aMD: accelerated molecular dynamics
IND: indol-3-pyruvate
