# Supplementary material for: Modulating D-amino acid oxidase (DAAO) substrate specificity through facilitated solvent access
Source: PLoS One. 2018 Jun 15;13(6):e0198990. doi: 10.1371/journal.pone.0198990 (PMC6003678; doi:10.1371/journal.pone.0198990)
Supplement: S1 Table — (PDF) [file pone.0198990.s001.pdf]

S. No	Origin	Location	Modification	Sequence
1	pkDAAO	First and Second Shell	Alanine mutation	L51A
2	pkDAAO	First and Second Shell	Alanine mutation	Q53A
3	pkDAAO	First and Second Shell	Alanine mutation	P54A
4	pkDAAO	First and Second Shell	Alanine mutation	Y55A
5	pkDAAO	First and Second Shell	Alanine mutation	T56A
6	pkDAAO	First and Second Shell	Alanine mutation	N96A
7	pkDAAO	First and Second Shell	Alanine mutation	N134A
8	pkDAAO	First and Second Shell	Alanine mutation	I215A
9	pkDAAO	First and Second Shell	Alanine mutation	H217A
10	pkDAAO	First and Second Shell	Alanine mutation	Y224A
11	pkDAAO	First and Second Shell	Alanine mutation	Y228A
12	pkDAAO	First and Second Shell	Alanine mutation	I230A
13	pkDAAO	First and Second Shell	Alanine mutation	F242A
14	pkDAAO	First and Second Shell	Alanine mutation	R283A
15	pkDAAO	First and Second Shell	Alanine mutation	G313A
16	pkDAAO	Wild type	Nil	Y55
17	pkDAAO	Y55	Mutated to C	Y55C
18	pkDAAO	Y55	Mutated to D	Y55D
19	pkDAAO	Y55	Mutated to E	Y55E
20	pkDAAO	Y55	Mutated to F	Y55F
21	pkDAAO	Y55	Mutated to G	Y55G
22	pkDAAO	Y55	Mutated to H	Y55H
23	pkDAAO	Y55	Mutated to I	Y55I
24	pkDAAO	Y55	Mutated to K	Y55K
25	pkDAAO	Y55	Mutated to L	Y55L
26	pkDAAO	Y55	Mutated to M	Y55M
27	pkDAAO	Y55	Mutated to N	Y55N
28	pkDAAO	Y55	Mutated to P	Y55P
29	pkDAAO	Y55	Mutated to Q	Y55Q
30	pkDAAO	Y55	Mutated to R	Y55R
31	pkDAAO	Y55	Mutated to S	Y55S
32	pkDAAO	Y55	Mutated to T	Y55T
33	pkDAAO	Y55	Mutated to V	Y55V
34	pkDAAO	Y55	Mutated to W	Y55W
35	pkDAAO	Active site ‘lid’ loop residues 223-225 (I223-Y224-N235)	Glycine mutations to lid loop residues	I223G-Y224G-N235G
36	pkDAAO	Active site ‘lid’ loop residues 223-225 (I223-Y224-N235), and Y55	Y55A in combination with glycine mutations to the lid loop residues	Y55A-I223G-Y224G-N235G
37	pkDAAO	Active site ‘lid’ loop residues I223-Y224, and Y55	Alanine mutation of the I223 residue	I223A
38	pkDAAO	Active site ‘lid’ loop residues I223-Y224, and Y55	Alanine mutations of I223 - Y224, and their combinations with Y55A mutation	Y55A-I223A
39	pkDAAO	Active site ‘lid’ loop residues I223-Y224, and Y55	Alanine mutations of I223 - Y224, and their combinations with Y55A mutation	Y55A-Y224A
40	pkDAAO	Active site ‘lid’ loop residues I223-Y224, and Y55	Alanine mutations of I223 and Y224	I223A-Y224A
41	pkDAAO	Active site ‘lid’ loop residues I223-Y224, and Y55	Alanine mutations of I223 - Y224, and their combinations with Y55A mutation	Y55A/I223A/Y224A
42	pkDAAO	Active site ‘lid’ loop deletion	‘Lid’ loop residues R221 and N225 joined (by deleting G222-I223-Y224 residues)	R221-N225
43	pkDAAO	Active site ‘lid’ loop deletion, and Y55	‘Lid’ loop residues R221 and N225 joined (by deleting G222-I223-Y224 residues), in combination with the Y55A mutation	Y55A--R221-N225
44	hDAAO	Active site ‘lid’ loop deletion	‘Lid’ loop residues R221 and N225 joined (by deleting G222-I223-Y224 residues)	R221-N225
45	hDAAO	Active site ‘lid’ loop deletion, and Y55	‘Lid’ loop residues R221 and N225 joined (by deleting G222-I223-Y224 residues), in combination with the Y55A mutation	Y55A--R221-N225
46	hDAAO	Wild type	Nil	Y55
47	hDAAO	Y55 position	Mutated to A	Y55A
48	hDAAO	Y55 position	Mutated to W	Y55W
49	pkDAAO	Y55 position and T56	Y55 mutated to A and T56 to L	Y55A-T56L
50	pkDAAO	Y55 position and T56	Y55 mutated to W and T56 to L	Y55W-T56L
51	hDAAO	Y55 position and L56	Y55 mutated to A and L56 to T	Y55A-L56T
52	hDAAO	Y55 position and L56	Y55 mutated to W and L56 to T	Y55W-L56T
