# Supplementary material for: Modulating D-amino acid oxidase (DAAO) substrate specificity through facilitated solvent access
Source: PLoS One. 2018 Jun 15;13(6):e0198990. doi: 10.1371/journal.pone.0198990 (PMC6003678; doi:10.1371/journal.pone.0198990)
Supplement: S2 Table — Freq–frequency of tunnels identified with probe radius 0.9 during 50 ns MD simulations; Avg B–average bottleneck radius [Å], Avg L–average length [Å]. (PDF) [file pone.0198990.s002.pdf]

Tunnel	pkDAAO			hDAAO			hDAAO Y55A			hDAAO Y55A L56T			hDAAO Y55A L56T D-Trp		
	Freq	Avg B	Avg L	Freq	Avg B	Avg L	Freq	Avg B	Avg L	Freq	Avg B	Avg L	Freq	Avg B	Avg L
T1	66.0	1.2 ± 0.2	16.4 ± 3.4	100	1.9 ± <0.1	12.0 ± 1.5	98.9	1.9 ± 0.2	10.0 ± 1.1	99.3	2.2 ± 0.3	11.5 ± 1.6	66.4	1.2 ± 0.2	13.8 ± 3.5
T2	66.7	1.2 ± 0.2	14.7 ± 4.0	69.4	1.1 ± 0.1	19.6 ± 4.5	39.1	1.4 ± <0.1	17.4 ± 6.5	92.5	1.5 ± 0.1	22.3 ± 5.4	38.7	1.1 ± 0.1	19.4 ± 3.5
T3	72.9	1.2 ± 0.2	10.7 ± 2.9	35.7	1.0 ± <0.1	14.3 ± 3.6	5.7	1.0 ± <0.1	16.8 ± 1.6	53.2	1.4 ± 0.3	16.5 ± 1.5	6.7	1.0 ± 0.1	23.0 ± 3.0
