# Supplementary material for: Modulating D-amino acid oxidase (DAAO) substrate specificity through facilitated solvent access
Source: PLoS One. 2018 Jun 15;13(6):e0198990. doi: 10.1371/journal.pone.0198990 (PMC6003678; doi:10.1371/journal.pone.0198990)
Supplement: S3 Table — Rows represent the DAAO variant, where P is pkDAAO and H is hDAAO. Columns represent the corresponding substrate used (D-Ala to D-Val). (PDF) [file pone.0198990.s003.pdf]

Variants	D-Ala	D-Arg	D-Asn	D-Asp	D-Cys	D-Gln	D-Glu	Gly	D-His	D-Ile	D-Leu	D-Lys	D-Met	D-Phe	D-Pro	D-Ser	D-Thr	D-Trp	D-Tyr	D-Val
P Y55A	0.9	2.6	0.1	0.2	2	0.3	0.3	0.1	1.4	1.1	0.6	0.1	10.6	17.1	8	0	0.1	1.8	15.2	0.2
P Y55R	0.4	1	0	0.7	1.2	0.2	0.3	0.2	1.2	0.5	0.6	0.1	7.1	13.8	5.3	0	0	3.3	7.7	0.7
P Y55N	1.1	1.6	0	0.4	1.5	0.3	0.4	0.2	1.2	0.6	0.4	0.1	7.7	13.8	11.4	0.1	0.2	2	9	0.4
P Y55D	0.8	2	0	0.3	1.2	0.1	0.2	0.1	1.1	0.5	0.2	0.1	5.4	8.6	8.8	0.1	0.1	0.8	4.1	0
P Y55C	0.7	1.3	0	0.2	0.9	0.1	0.2	0.2	0.9	0.8	0.7	0.1	5.7	6.5	4.6	0.2	0.1	0.4	0.7	0.2
P Y55Q	2.4	1.9	0.1	0.1	3.4	0.2	0.1	0.1	2.2	2.3	1.3	0.1	9.4	10.8	6.3	0.1	0.1	0.9	6.7	0.7
P Y55E	0.6	2.7	0	0.3	0.8	0.1	0.1	0	0.9	0.7	0.5	0.1	6.6	6.6	4.4	0.2	0.2	0.1	4.4	0.3
P Y55G	1.4	2	0	0.1	1.4	0.1	0.2	0	1.5	0.9	0.7	0.1	8.4	14.6	8.6	0.2	0.1	2.7	12.9	0.4
P Y55H	1.8	2	0	0.1	2.1	0.1	0.1	0	1.7	0.9	0.7	0	10.4	13.5	6.8	0.2	0	2.6	14.7	0.3
P Y55I	2.3	0.9	0.3	0.4	4.6	0.4	0.3	0.2	1.8	3.4	4.1	0.3	15.2	15.9	10.1	0.3	0.2	1.8	9.7	1.3
P Y55L	4.1	1.2	0	0.1	3.5	0.2	0.1	0	2.6	2.8	1.8	0.1	14.5	16.5	13.1	0.3	0.1	2.4	13.8	1.8
P Y55K	0.9	0.9	0	0.1	1.5	0.1	0.1	0	1.7	0.7	0.6	0	6.8	8.9	4.9	0.1	0	3	9.2	0.2
P Y55M	3.2	2.4	0.1	0.1	3.2	0.3	0.1	0	2.5	2.1	1.5	0.1	8.5	10	9.5	0.2	0	0.2	7.6	0.9
P Y55F	3.2	3.8	0	0.3	0.9	0.2	0.3	0.1	1.9	2.2	1.9	0	7	2.4	3.7	0.9	1.5	0.1	0.6	0.9
P Y55P	3.8	1.8	0	0.2	3.5	0.5	0.1	0	4.6	2.4	2.3	0	16.9	23.7	14.1	0.1	0.1	3.3	27.8	0.7
P Y55S	1.7	1.6	0.1	0.1	1.3	0.1	0.1	0	1.4	0.6	0.5	0	8.3	13.3	8.1	0.1	0.1	2	16.4	0.2
P Y55T	1.5	0.8	0	0	0.7	0.1	0.1	0	1.8	0.8	0.9	0	9.1	14.5	7.6	0.2	0.2	2	16.1	0.3
P Y55W	0.9	3	0.5	0.3	1.7	0.5	0.6	0.7	1	1	1.3	0.5	8.1	10	6.2	0.7	0.2	1.6	4.7	0.9
P Wildtype	4.1	1.1	0	0.3	2.9	0.1	0.1	0	0.8	3.7	1.7	0	7.2	9	13.5	2.4	0.3	0.9	5	2.4
P Y55V	1.2	0.8	0	0.1	1.9	0.1	0	0	0.8	1	1.4	0	8.5	11.4	5.9	0.3	0.3	1.5	14.3	0.4
P T56L	2.93	0.78	0.32	0.12	2.27	0.86	0.10	0.03	1.15	3.31	1.12	0.44	5.62	10.43	12.27	3.48	0.64	3.08	6.30	2.16
P Y55A/T56L	2.61	0.83	0.21	0.11	2.96	0.29	0.33	0.06	1.83	0.79	0.90	0.27	10.53	18.96	7.77	0.49	0.14	2.44	11.76	0.48
P Y55W/T56L	4.40	2.15	0.45	0.10	3.62	0.50	0.05	0.10	0.98	3.03	1.50	0.17	10.14	16.14	9.92	0.81	0.20	1.76	9.21	1.08
H Wildtype	4.27	1.00	0.87	0.22	3.14	1.06	0.20	0.10	2.80	6.05	3.40	0.27	5.21	11.05	12.32	2.06	0.50	3.41	6.76	2.68
H Y55A	4.06	2.40	2.18	0.06	3.50	1.74	0.07	0.03	8.25	4.10	4.92	0.39	10.41	23.01	10.35	1.65	0.30	7.23	15.94	3.71
H Y55W	3.46	2.15	1.57	0.06	4.60	1.14	0.13	0.24	5.87	3.15	4.77	0.46	7.10	18.64	7.65	1.11	0.16	3.67	11.08	3.91
H L56T	5.47	0.99	0.69	0.07	3.22	0.61	0.09	0.10	2.34	4.86	3.82	0.28	4.90	11.35	9.61	1.45	0.29	2.41	4.54	3.19
H Y55A/L56T	2.58	2.36	1.33	0.08	4.60	1.08	0.07	0.07	3.95	3.06	3.34	0.20	6.08	18.46	7.90	0.79	0.21	4.45	11.36	2.53
H Y55W/L56T	2.43	2.17	0.97	0.35	3.56	0.72	0.09	0.23	3.40	2.31	4.00	0.45	5.76	11.60	5.79	0.69	0.35	3.04	5.53	2.20
