# Supplementary figures and images for: Modulating D-amino acid oxidase (DAAO) substrate specificity through facilitated solvent access
Source: PLoS One. 2018 Jun 15;13(6):e0198990. doi: 10.1371/journal.pone.0198990 (PMC6003678; doi:10.1371/journal.pone.0198990)

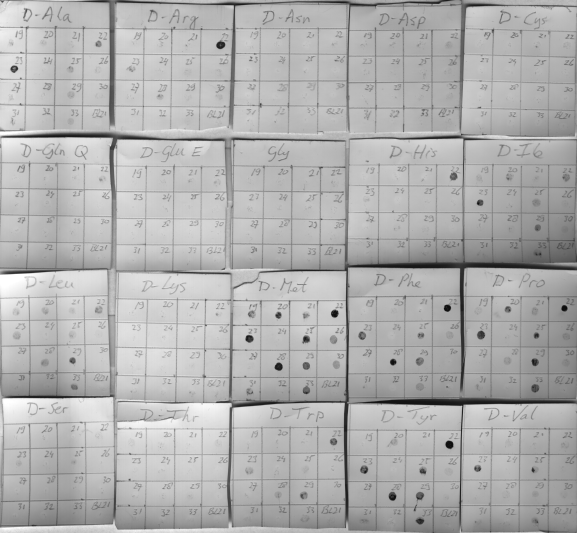

Supplement: S1 Fig — The variants 1–15 from S1 Table were designed by mutating the first and second shell residues of pkDAAO to an alanine. Each panel in S1 Fig represents the activity of all the variants towards a specific substrate (D-Ala, D-Arg, A-Asn, to D-Val). Equal amounts of the BL21 host cell (that lack the expression vector, but similarly induced) crude lysates were blotted alongside to observe any background activity. The numbers mentioned beside the corresponding blotted samples (19–33) represent the following alanine mutants, where the variant numbers in S1 Table are shown within parenthesis. 19: L51A (1), 20: Q53A (2), 21: P54A (3), 22: Y55A (4), 23: T56A (5), 24: N96A (6), 25: N134A (7), 26: I215A (8), 27: H217A (9), 28: Y224A (10), 29: Y228A (11), 30: I230A (12), 31: F242A (13), 32: R283A (14), 33: G313A (15). (TIF) [file pone.0198990.s004.tif]

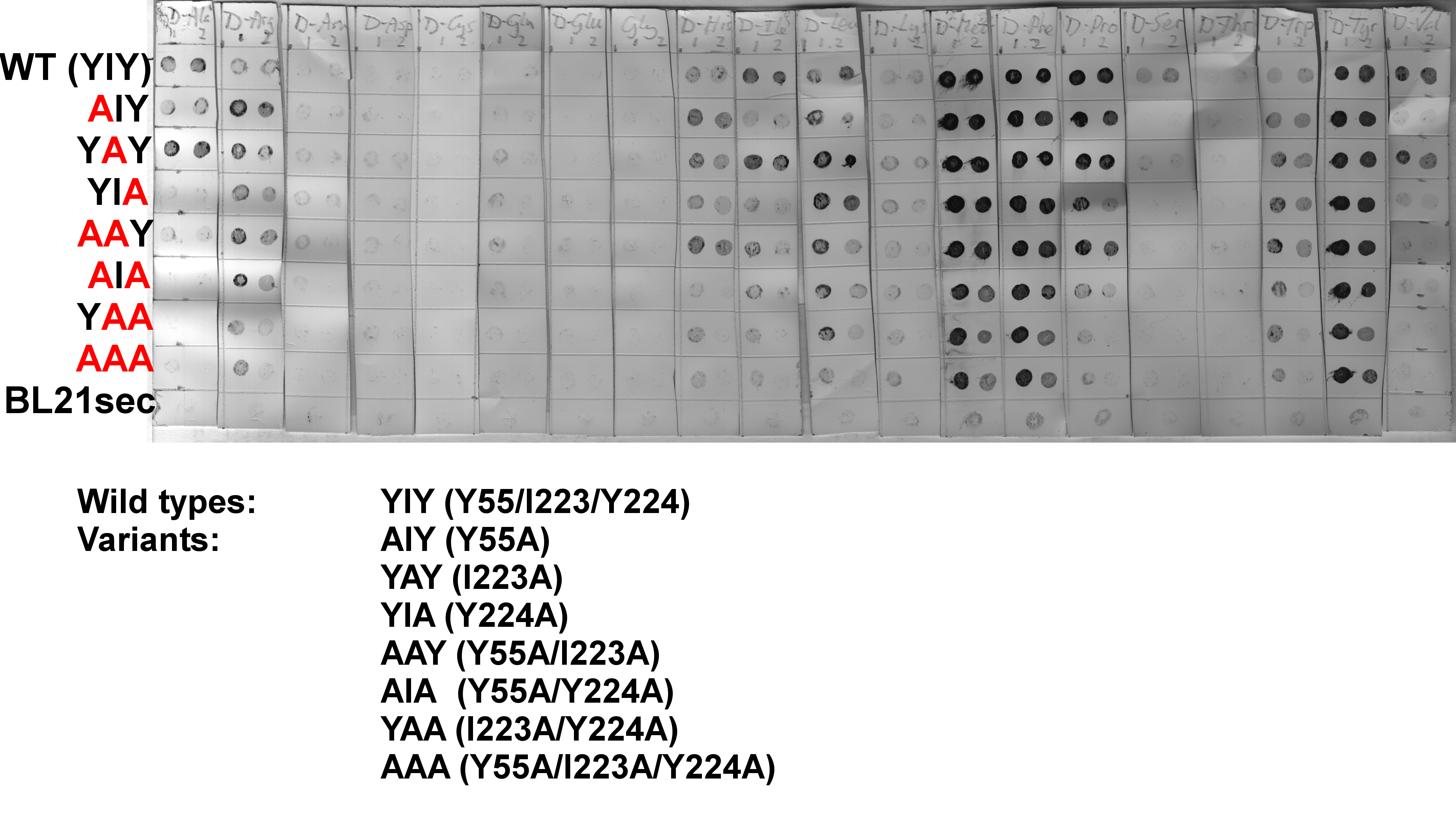

Supplement: S4 Fig — Alongside the pkDAAO variants, in duplicate, an equal amount of the BL21 host cell (that lacks the expression vector, but similarly induced) was blotted to observe any background activity. The rows represent each variant screened, where the wild type, Y55-I223-Y224, is YIY, and the corresponding alanine mutation is denoted in red. Columns represent the substrate used to screen the activity of the variants, from D-Ala to D-Val. (TIF) [file pone.0198990.s007.tif]

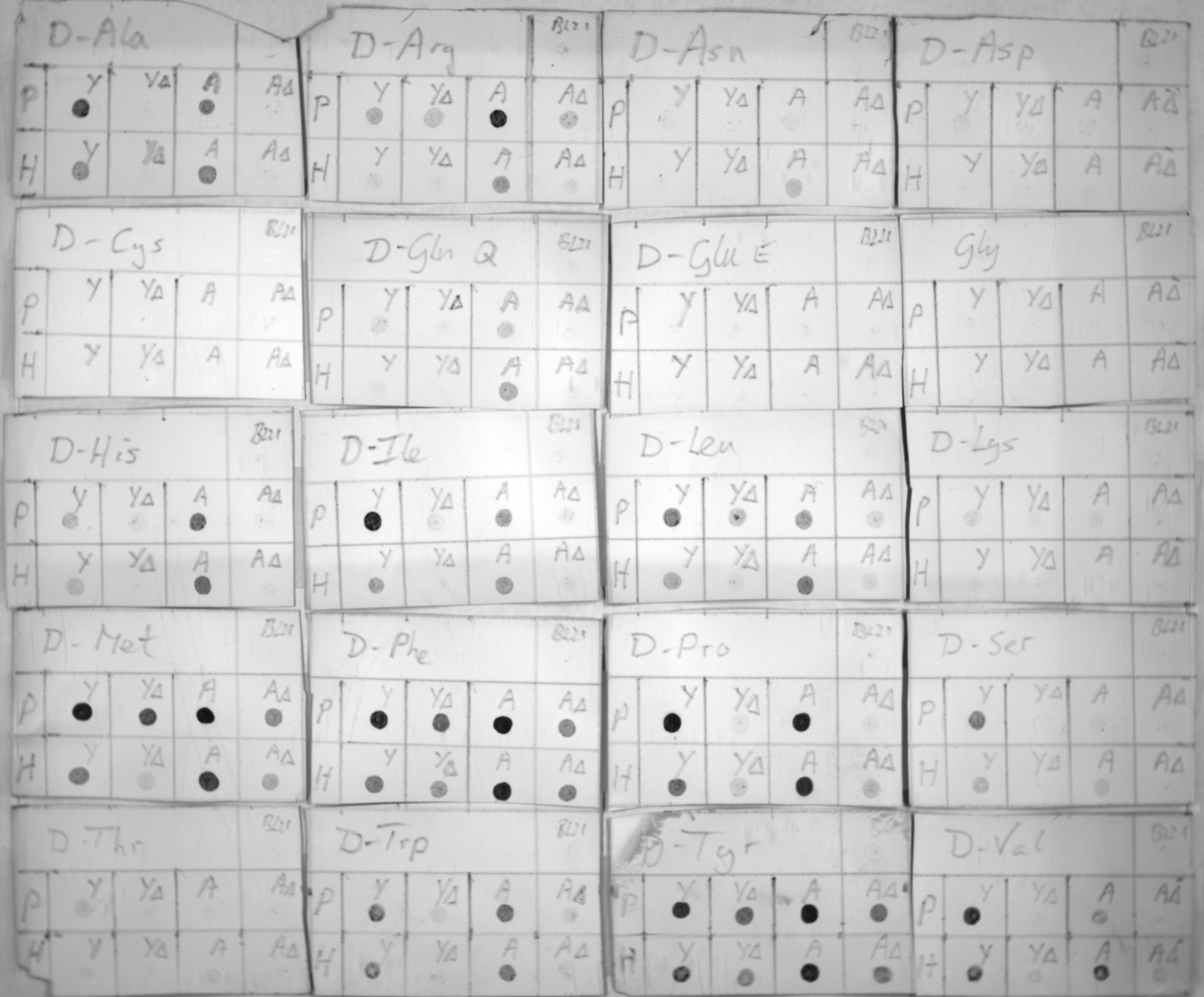

Supplement: S5 Fig — Crude cell lysates were blotted on the membrane, and the activities towards 19 D-amino acids and glycine substrates were analysed. Every panel in the figure represents the activity of the variants towards a specific substrate (D-Ala to D-Val), and BL21 host cells were dot-blotted as a control to compare background activity. Rows with either P or H denote the pkDAAO or hDAAO, respectively. In the columns, the following representation is followed: Y is the wild type, YΔ is the loop deletion mutant, A is the Tyr55Ala mutant, and AΔ is the double mutant involving both Tyr55Ala mutation and loop deletion. (TIF) [file pone.0198990.s008.tif]

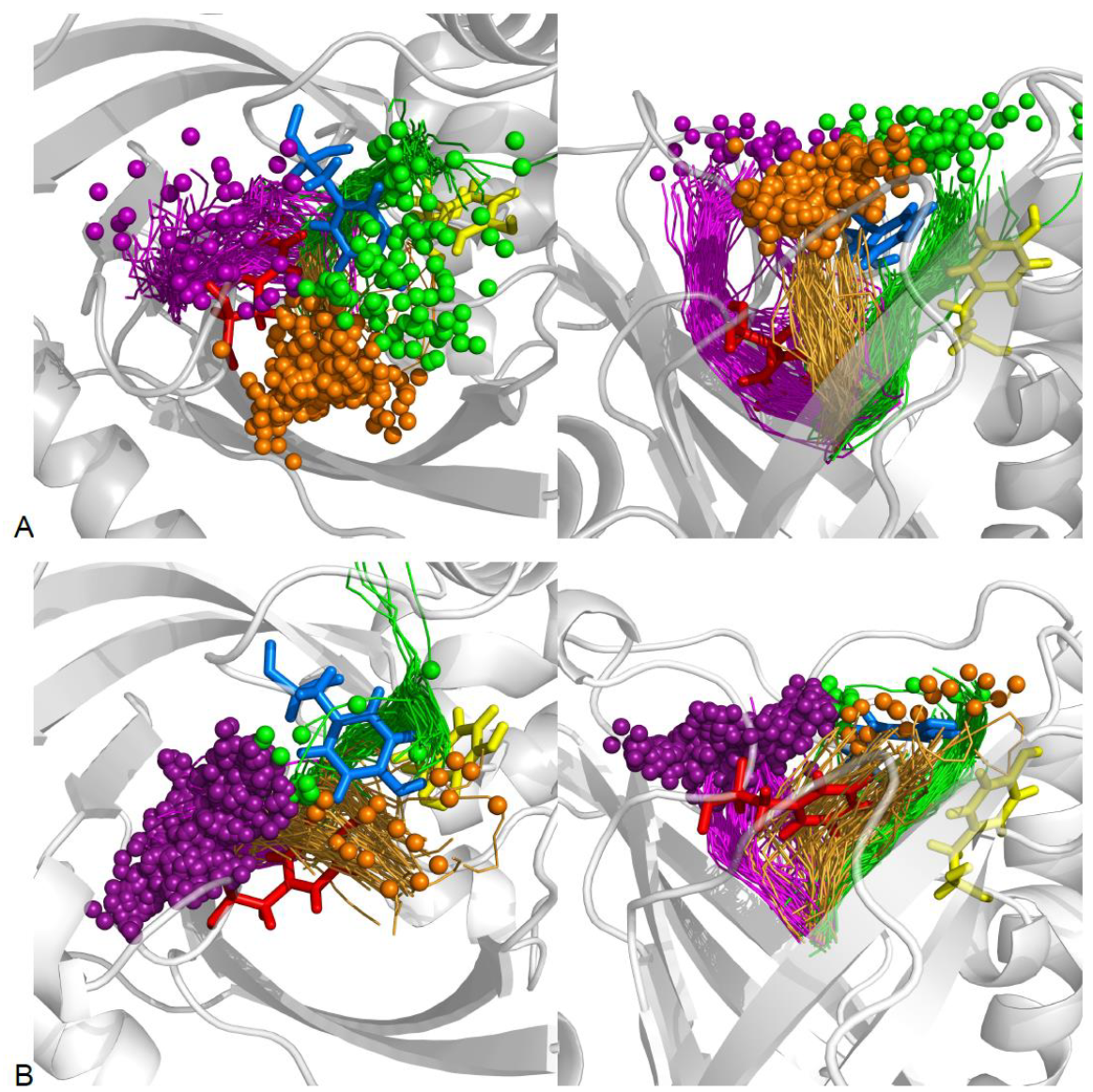

Supplement: S6 Fig — Comparison of tunnels detected by CAVER (represented by lines) and water inlets (represented by small spheres) detected by AQUA-DUCT in pkDAAO wild type (A) and hDAAO wild type (B). Residues dividing tunnels are represented by stick (Y55 blue, Y224 red, Y314 yellow), protein by semitransparent cartoon), Panel on the right shows same region of the protein rotated approximately 90°. (TIF) [file pone.0198990.s009.tif]

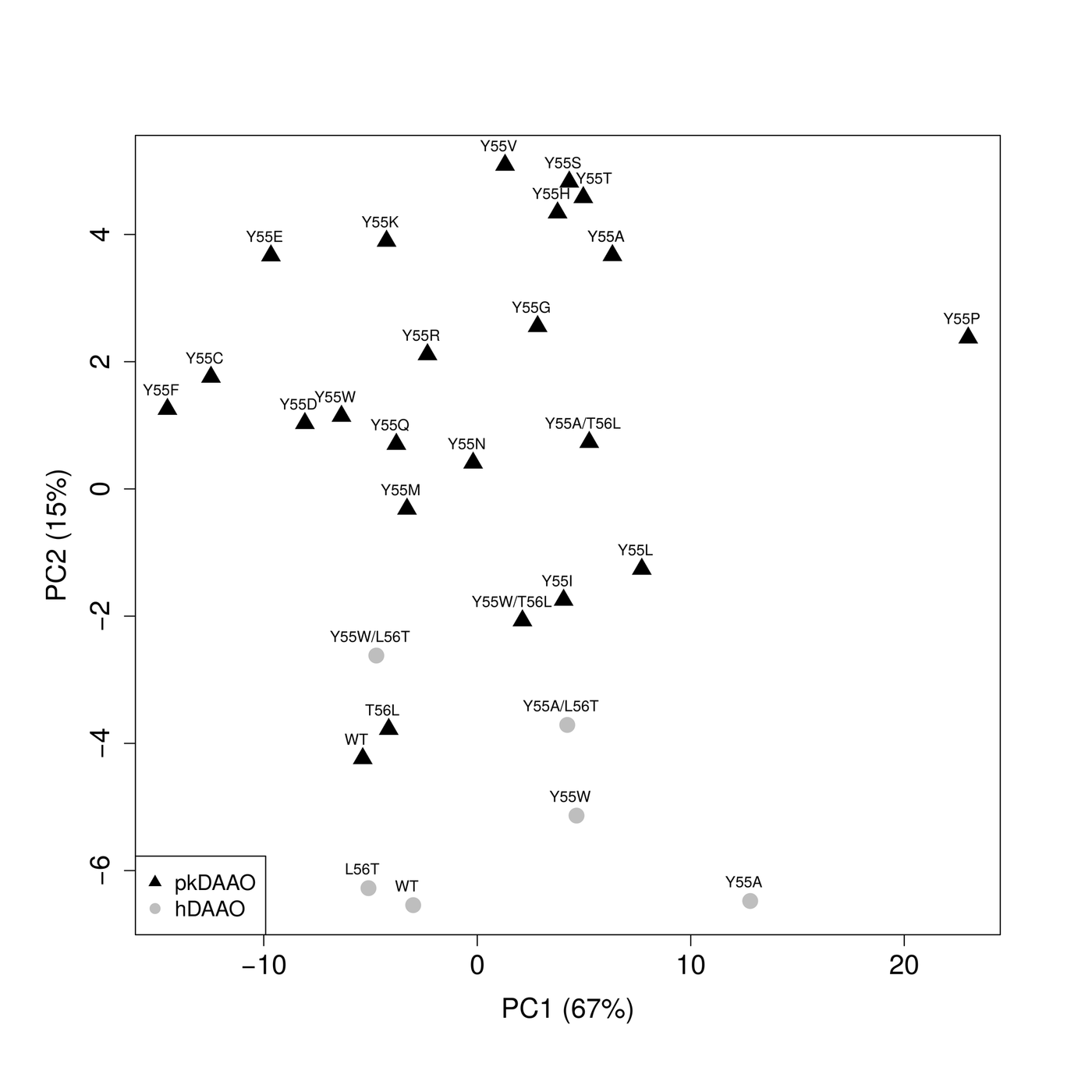

Supplement: S7 Fig — (TIF) [file pone.0198990.s010.tif]
